# Supplementary material for: FLT1 activation in cancer cells promotes PARP-inhibitor resistance in breast cancer
Source: EMBO Mol Med. 2024 Jul 2;16(8):8. doi: 10.1038/s44321-024-00094-2 (PMC11319505; doi:10.1038/s44321-024-00094-2)
Supplement: Supplementary file 1 — Appendix [file 44321_2024_94_MOESM1_ESM.pdf]

Table of Contents

Appendix Figure S1..2

Appendix Figure S2..3

Appendix Table S1..4

Appendix Figure S1

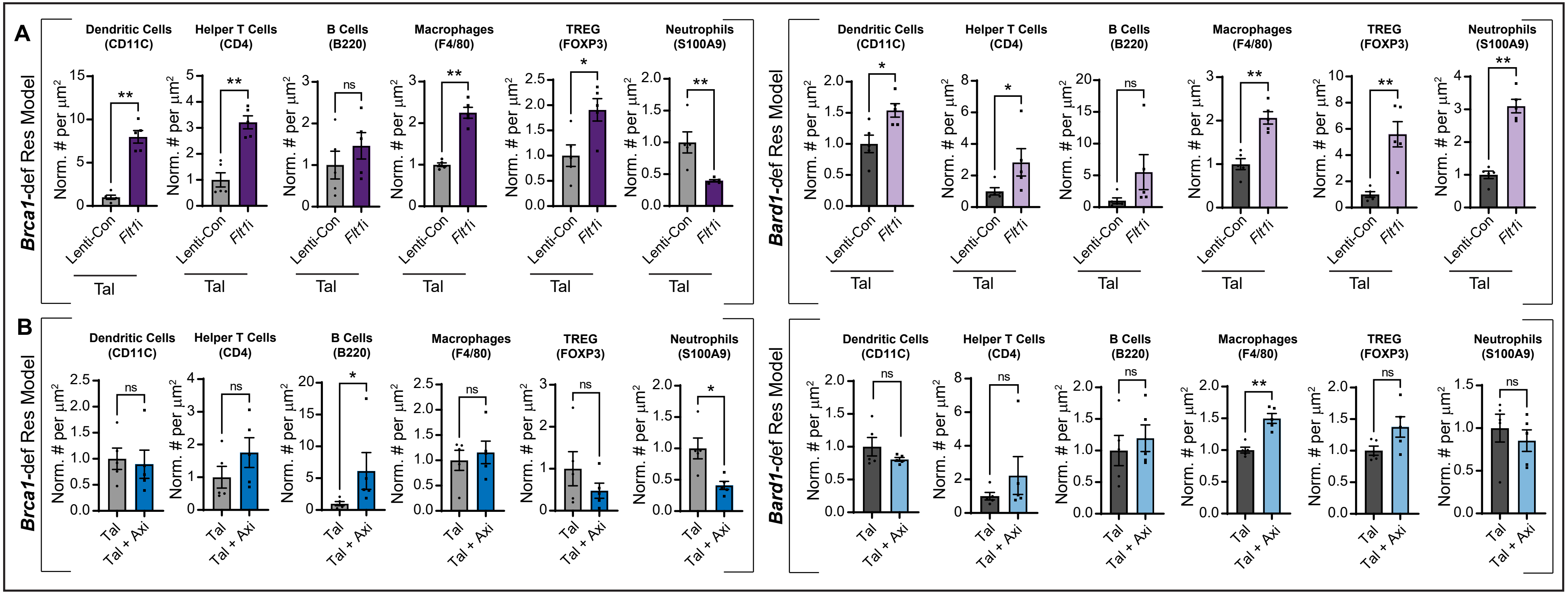

Appendix Figure S1. Immunohistochemical analysis of the immune cells in the tumor microenvironment following FLT1 blockade and talazoparib treatment in mouse models. A, Multiple immune markers were analyzed in talazoparib-resistant (“Res”) tumor sections from both Brca1-def and Bard1-def breast cancer models expressing either Lenti-Con or Flt1i and treated with talazoparib (“Tal”) (see Fig. 3C). *n* = 5 for both groups from each model. Data are presented as mean values ± SEM. *P* values were determined by a two-tailed, unpaired, Mann–Whitney test and are listed in parentheses following the immunostains analyzed: for the *Brca1*-def model, CD11C (0.0079), CD4 (0.0079), B220 (ns), F4/80 (0.0079), FOXP3 (0.0317), and S100A9 (0.0079) and for the Bard1-def model, CD11C (0.0317), CD4 (0.0317), B220 (ns), F4/80 (0.0079), FOXP3 (0.0079), and S100A9 (0.0079). ns: not significant. **B**, Multiple immune markers were analyzed in Res tumors from both *Brca1*-def and *Bard1*-def breast cancer models treated with Tal or Tal + axitinib (“Axi”) (see Fig. 4A/E). *n* = 5 for both groups from each model. Data are presented as mean values ± SEM. *P* values were determined by a two-tailed, unpaired, Mann–Whitney test. ns: not significant. For the *Brca1*-def model, * indicates *P* = 0.0159 for both quantitation for B220 and S100A9. For the *Bard1*-def model, ** indicates *P* = 0.0079 for F4/80. Remaining comparisons for other immune cells were not significant (ns).

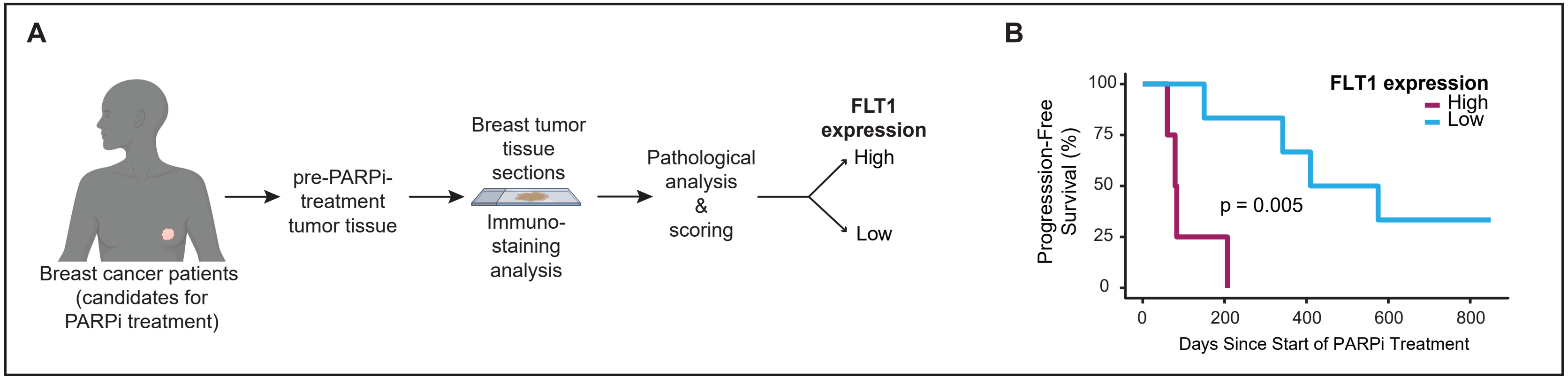

Appendix Figure S2. High FLT1 expression in human tumor cells prior to PARPi treatment is associated with shorter progression-free survival in breast cancer patients. A, Schematic representation of the workflow for the pathological evaluation of the FLT1 expression in human tumor cells in breast tumor sections pre-PARPi treatment in breast cancer patients. FLT1 immunostainings were performed on tumor tissue specimens (biopsies/resected material) from 10 patients with breast cancer and were obtained prior to PARPi treatment. The immunostained samples were scored by a pathologist who was blinded to the sample details, as either FLT1-high or -low expression. **B**, Kaplan–Meier plots for the PFS of patients described in **A**. Data were analyzed using the log-rank test: $\chi 2$ = 8.044, degrees of freedom (d.f.) = 1; P = 0.005; n = 10 patients.

Appendix Table S1

Sample ID	Germline Variant	pFLT1
PARP 007	BRCA1	High
PARP 12	BRCA1	Low
PARP 21	BRCA1	Low
PARP 50	BRCA2	Low
S19-30556	PALB2	Low
S19-46410	BRCA2	High
S19-713	BRCA2	Low
S18-73798	BRCA2	High
S19-15838	BRCA1	High
S17-58773	BRCA2	High

Appendix Table S1.
De-identified list of tumor tissue samples from human breast cancer patients (n = 10) with confirmed *BRCA1/2* or *PALB2* germline mutations, along with high or low levels of pFLT1. Samples were collected prior to PARP inhibitor treatment.
