# Supplementary figures and images for: FLT1 activation in cancer cells promotes PARP-inhibitor resistance in breast cancer
Source: EMBO Mol Med. 2024 Jul 2;16(8):8. doi: 10.1038/s44321-024-00094-2 (PMC11319505; doi:10.1038/s44321-024-00094-2)

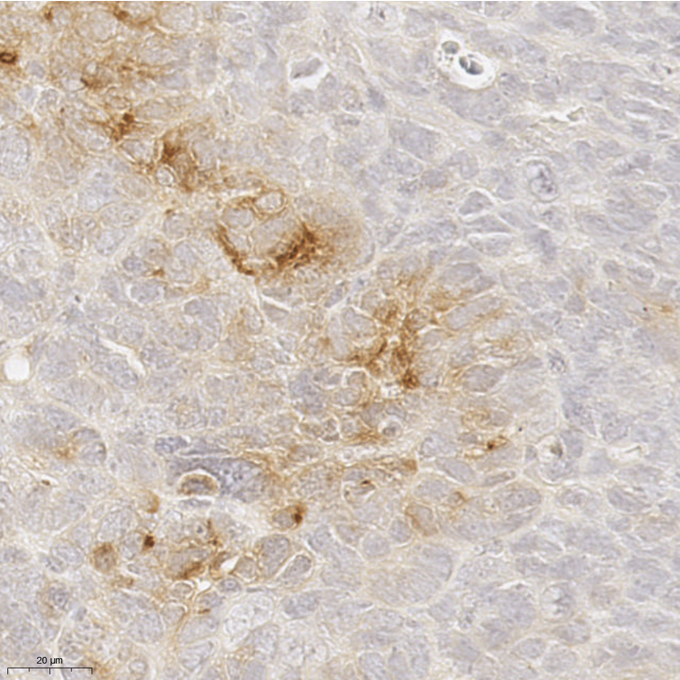

Supplement: Supplementary file 4 — Source data Fig. 2 [file 44321_2024_94_MOESM4_ESM.zip › Figure 2/2E Image Data micr. image/Brca1-def Sen.tif]

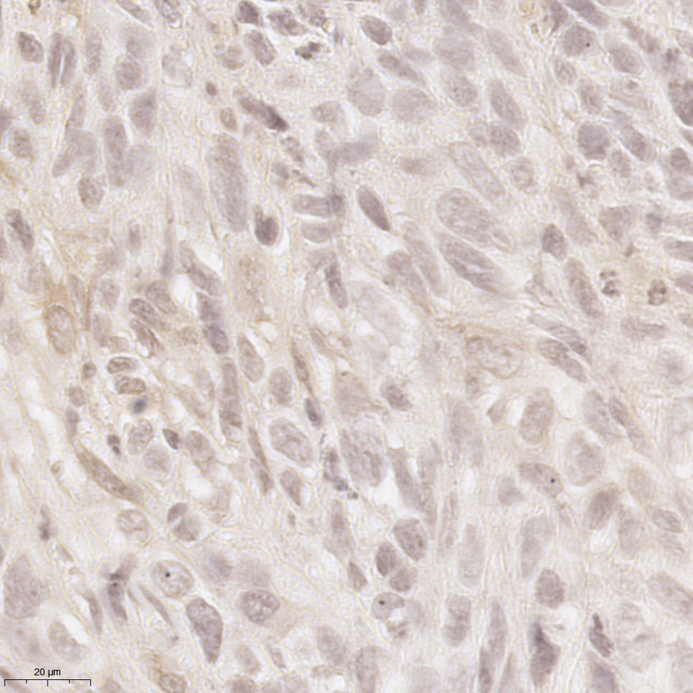

Supplement: Supplementary file 4 — Source data Fig. 2 [file 44321_2024_94_MOESM4_ESM.zip › Figure 2/2E Image Data micr. image/Bard1-def Sen.tif]

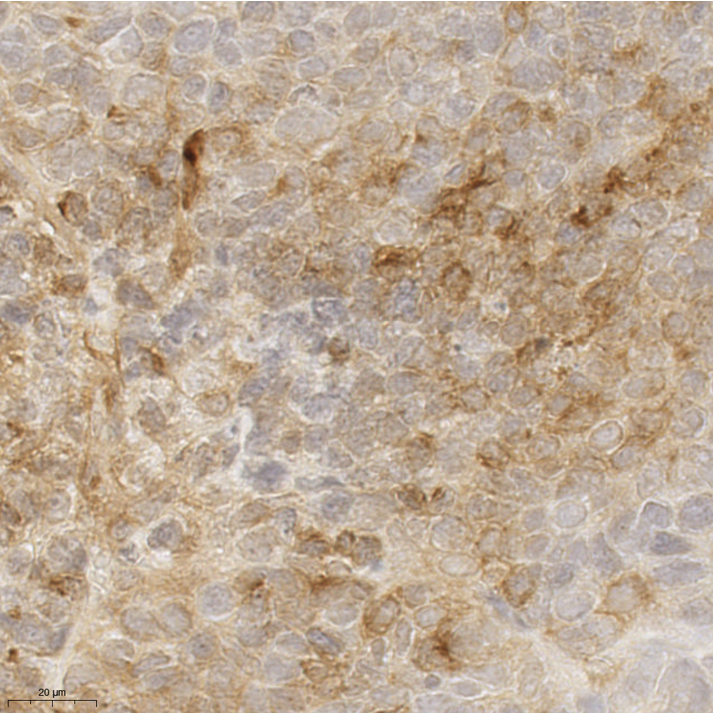

Supplement: Supplementary file 4 — Source data Fig. 2 [file 44321_2024_94_MOESM4_ESM.zip › Figure 2/2E Image Data micr. image/Brca1-def Res.tif]

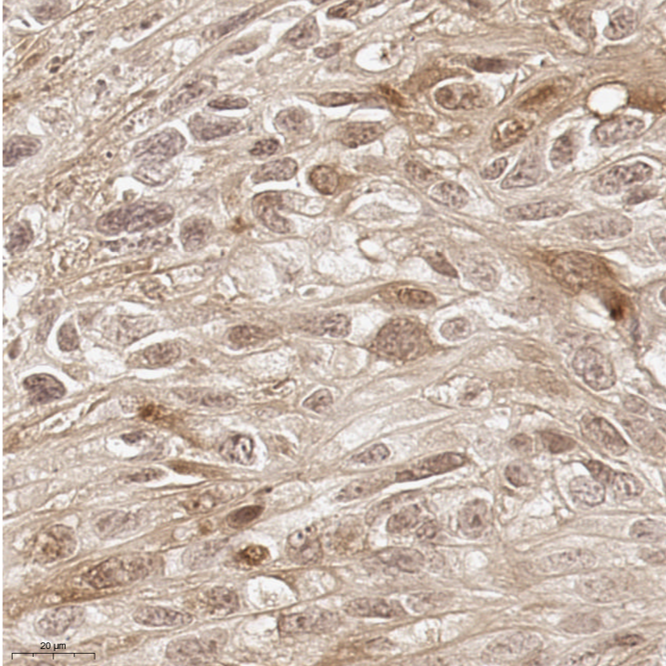

Supplement: Supplementary file 4 — Source data Fig. 2 [file 44321_2024_94_MOESM4_ESM.zip › Figure 2/2E Image Data micr. image/Bard1-def Res.tif]

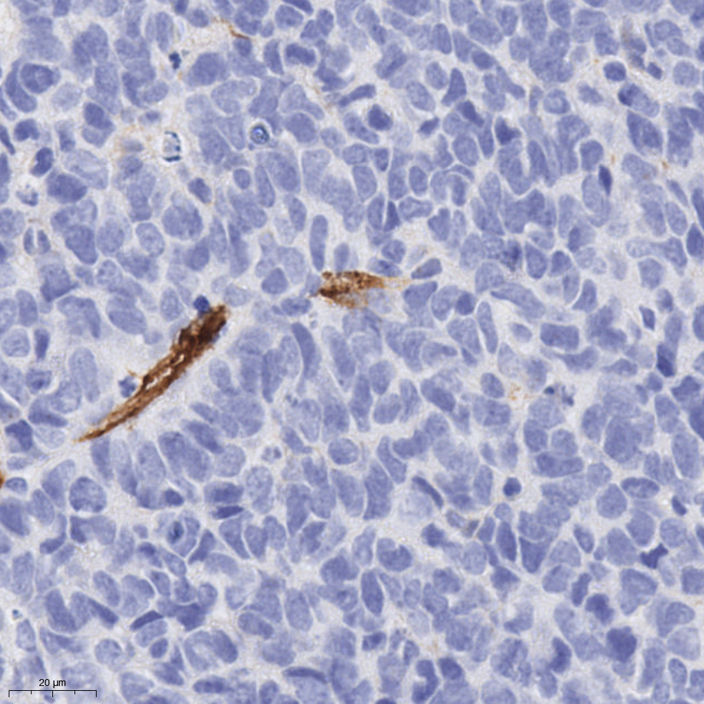

Supplement: Supplementary file 4 — Source data Fig. 2 [file 44321_2024_94_MOESM4_ESM.zip › Figure 2/2A Image Data micr. image/Brca1-def Sen.tif]

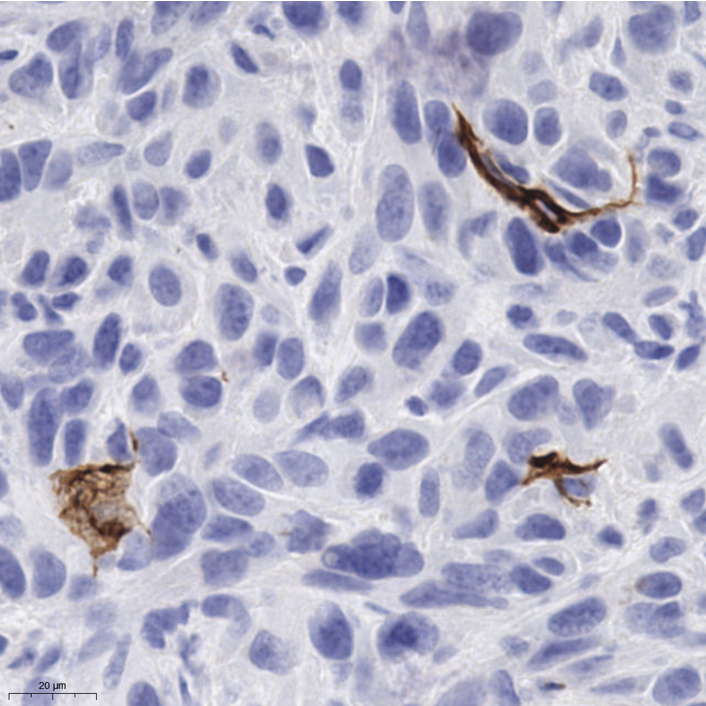

Supplement: Supplementary file 4 — Source data Fig. 2 [file 44321_2024_94_MOESM4_ESM.zip › Figure 2/2A Image Data micr. image/Bard1-def Sen.tif]

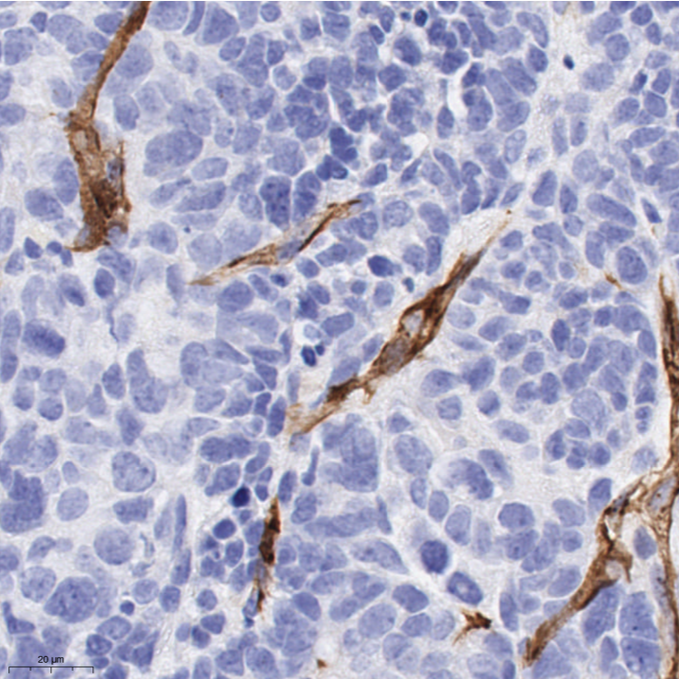

Supplement: Supplementary file 4 — Source data Fig. 2 [file 44321_2024_94_MOESM4_ESM.zip › Figure 2/2A Image Data micr. image/Brca1-def Res.tif]

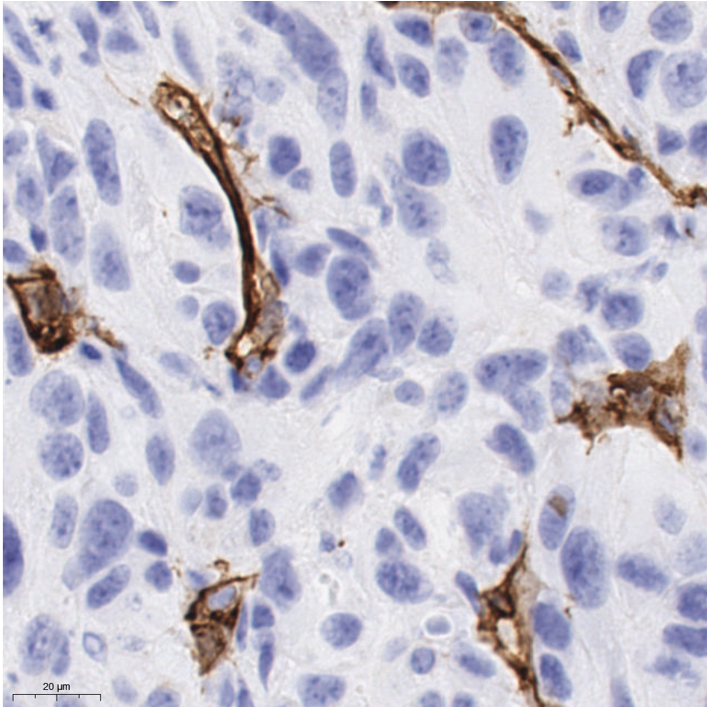

Supplement: Supplementary file 4 — Source data Fig. 2 [file 44321_2024_94_MOESM4_ESM.zip › Figure 2/2A Image Data micr. image/Bard1-def Res.tif]

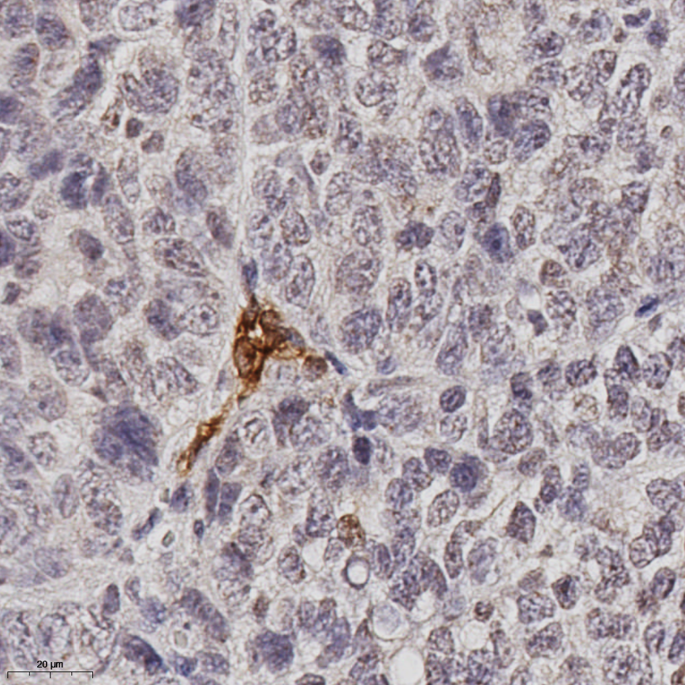

Supplement: Supplementary file 4 — Source data Fig. 2 [file 44321_2024_94_MOESM4_ESM.zip › Figure 2/2G Image Data micr. image/Brca1-def Sen.tif]

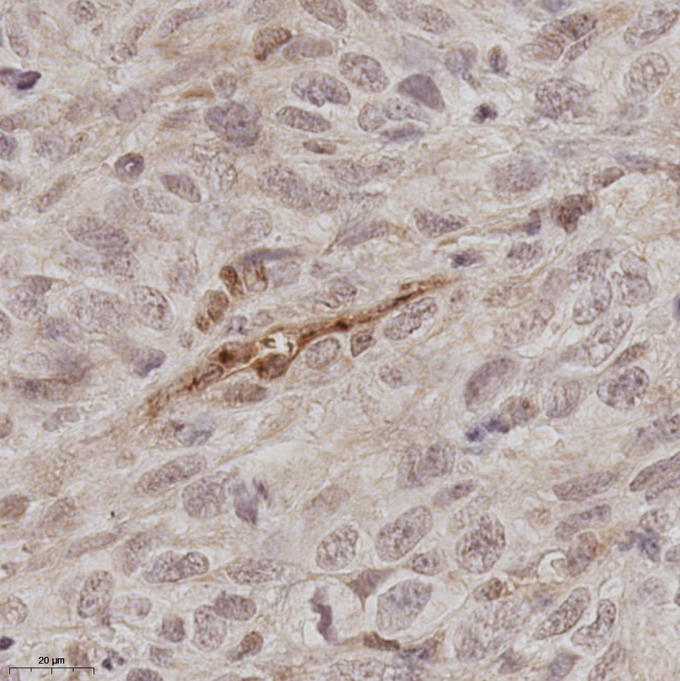

Supplement: Supplementary file 4 — Source data Fig. 2 [file 44321_2024_94_MOESM4_ESM.zip › Figure 2/2G Image Data micr. image/Bard1-def Sen.tif]

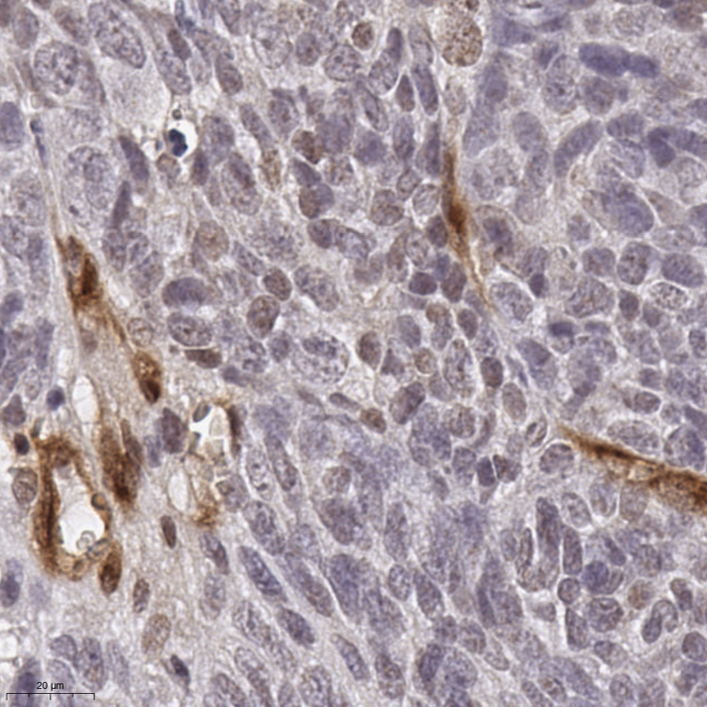

Supplement: Supplementary file 4 — Source data Fig. 2 [file 44321_2024_94_MOESM4_ESM.zip › Figure 2/2G Image Data micr. image/Brca1-def Res.tif]

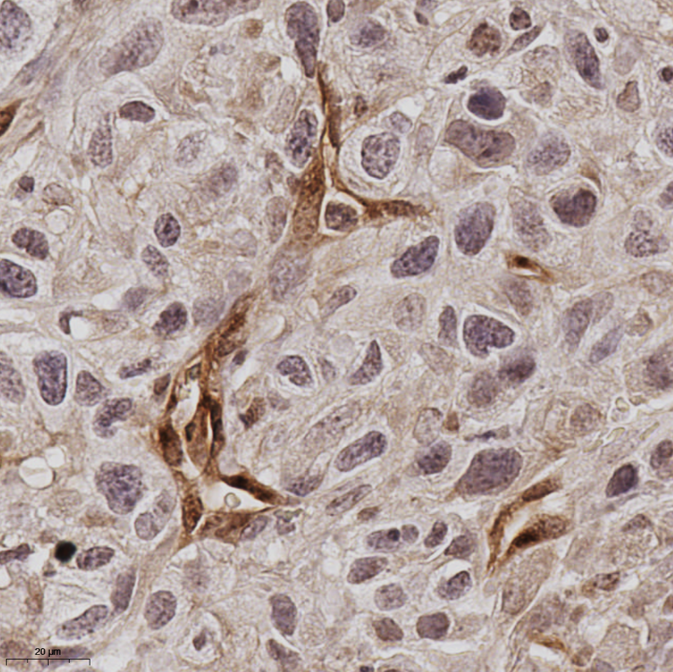

Supplement: Supplementary file 4 — Source data Fig. 2 [file 44321_2024_94_MOESM4_ESM.zip › Figure 2/2G Image Data micr. image/Bard1-def Res.tif]

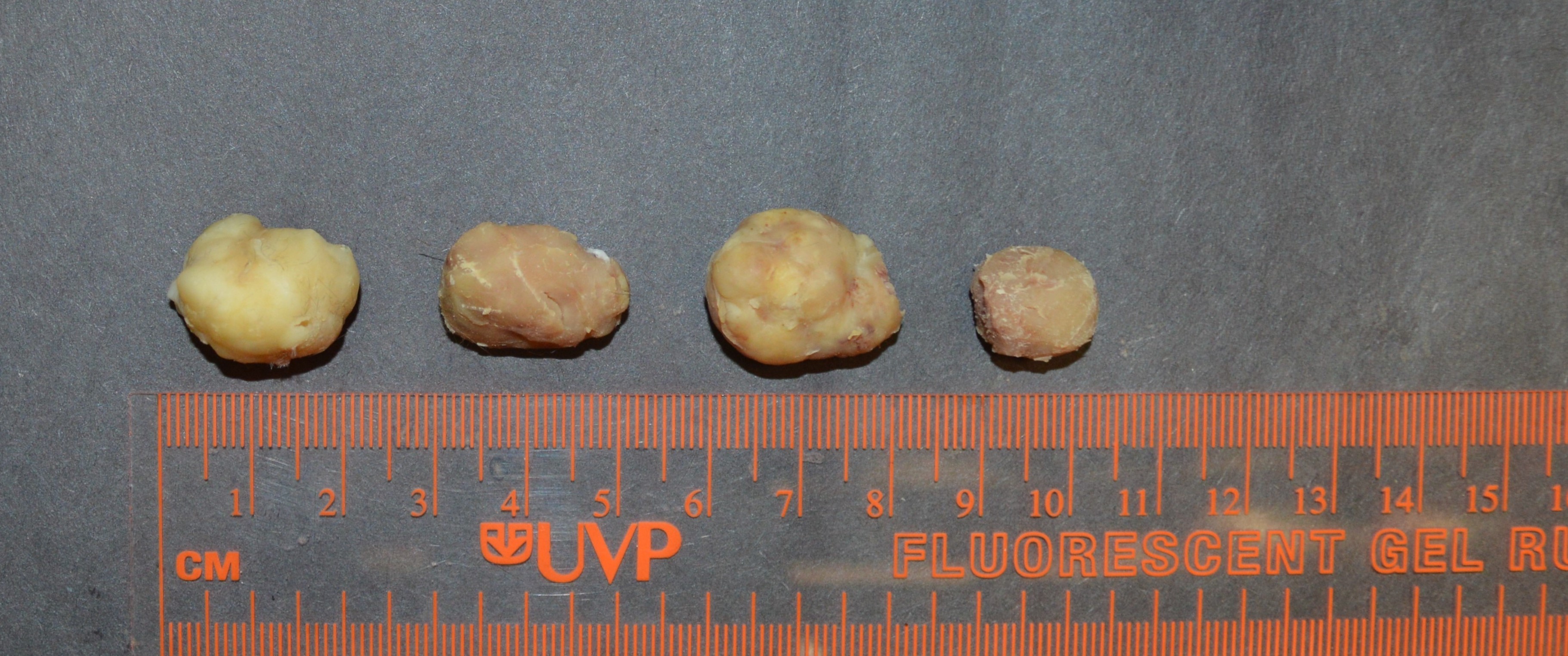

Supplement: Supplementary file 4 — Source data Fig. 2 [file 44321_2024_94_MOESM4_ESM.zip › Figure 2/2K Image Data whole mount/Brca1-def Tumors.tif]

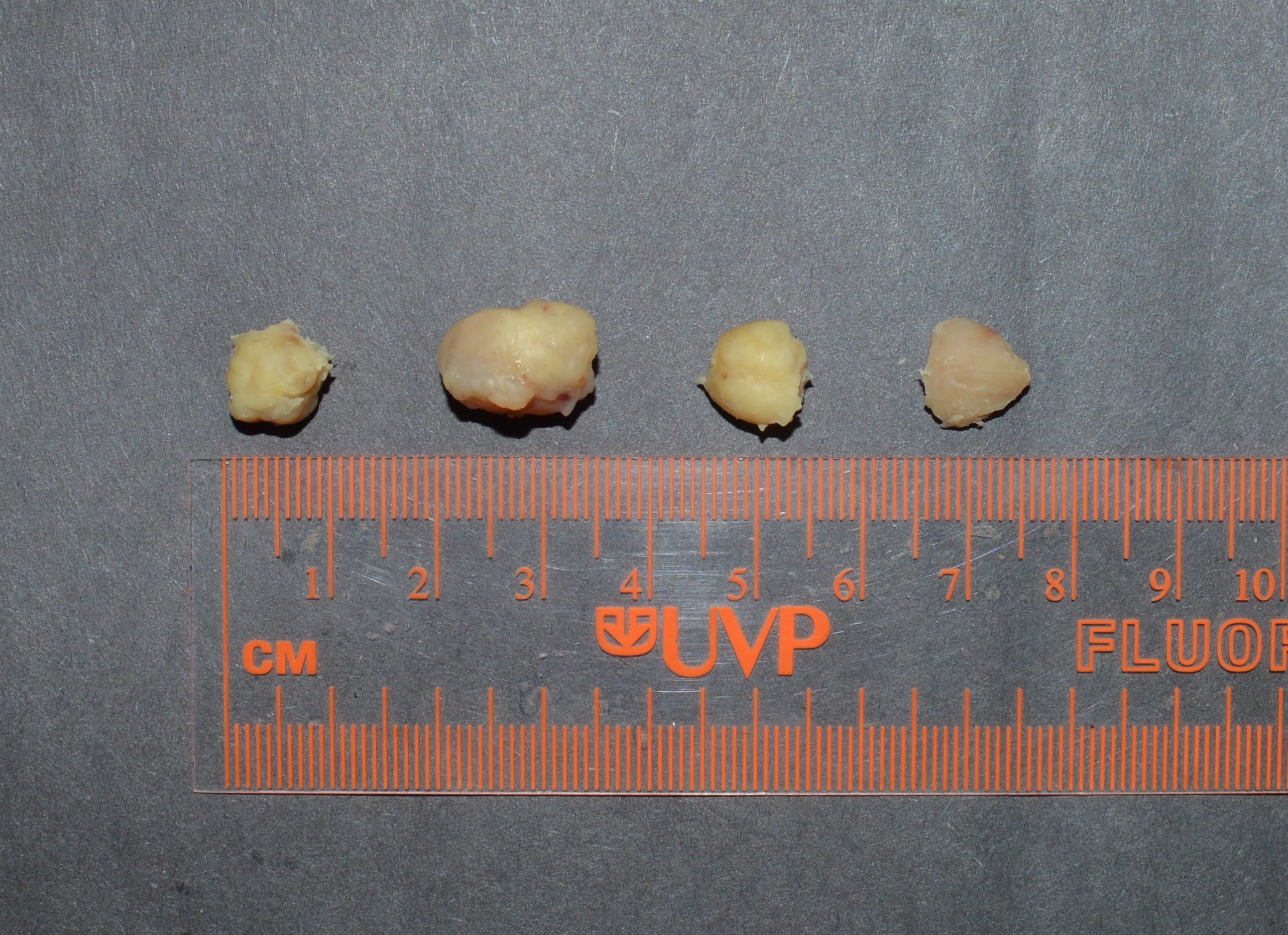

Supplement: Supplementary file 4 — Source data Fig. 2 [file 44321_2024_94_MOESM4_ESM.zip › Figure 2/2K Image Data whole mount/Bard1-def Tumors.tif]

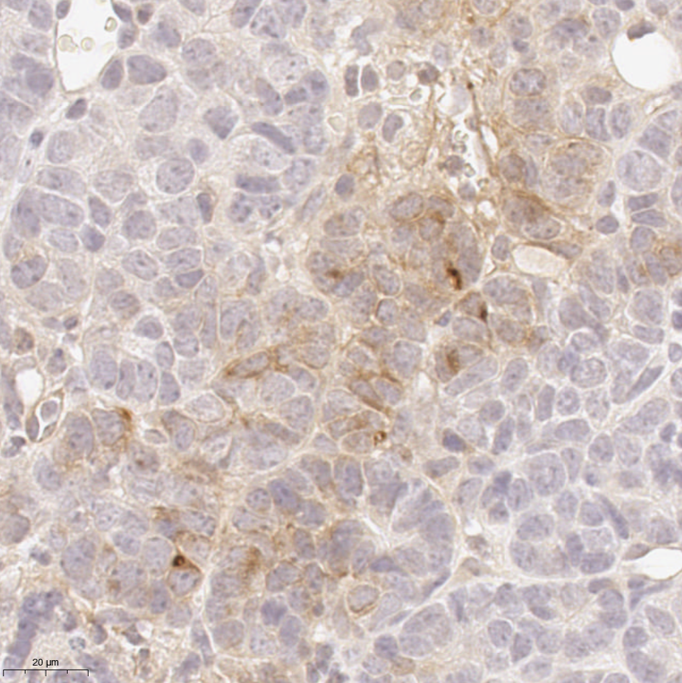

Supplement: Supplementary file 4 — Source data Fig. 2 [file 44321_2024_94_MOESM4_ESM.zip › Figure 2/2C Image Data micr. image/Brca1-def Sen.tif]

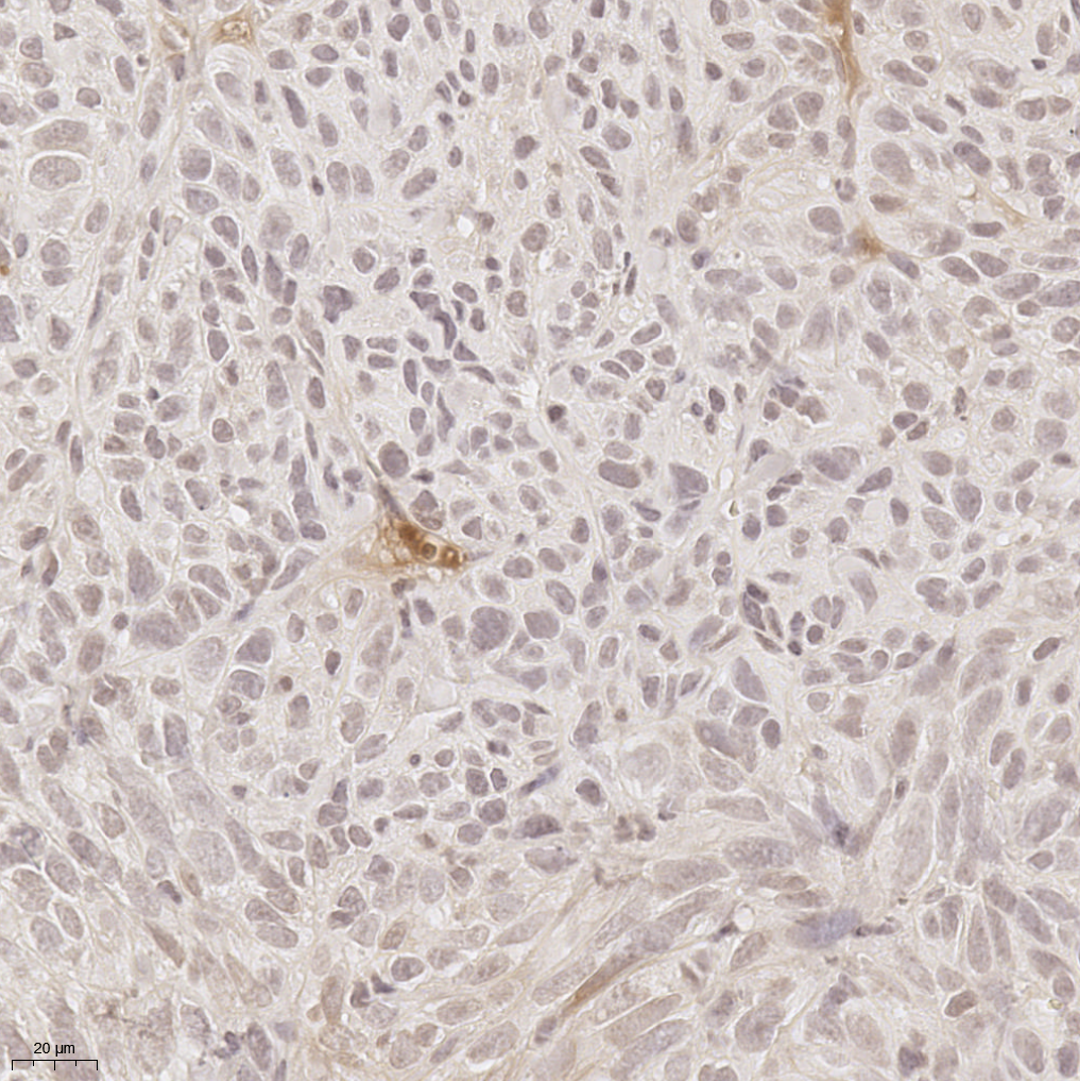

Supplement: Supplementary file 4 — Source data Fig. 2 [file 44321_2024_94_MOESM4_ESM.zip › Figure 2/2C Image Data micr. image/Bard1-def Sen.tif]

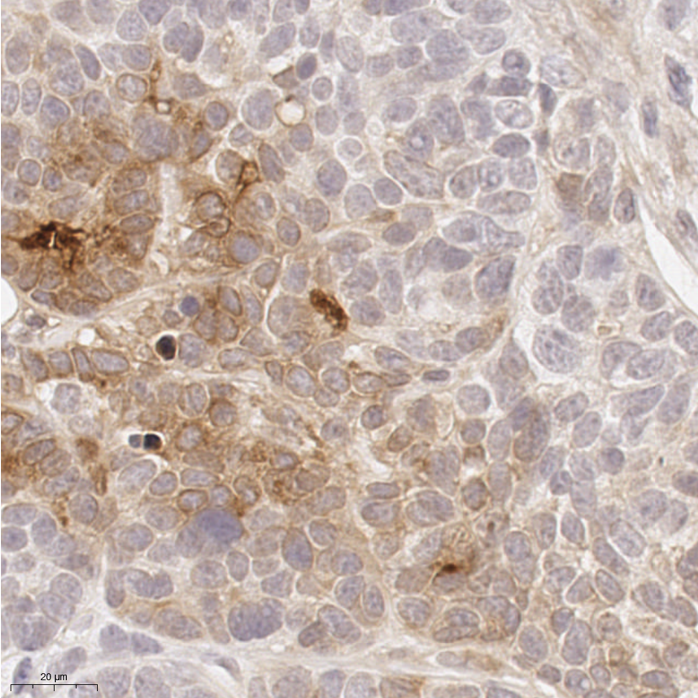

Supplement: Supplementary file 4 — Source data Fig. 2 [file 44321_2024_94_MOESM4_ESM.zip › Figure 2/2C Image Data micr. image/Brca1-def Res.tif]

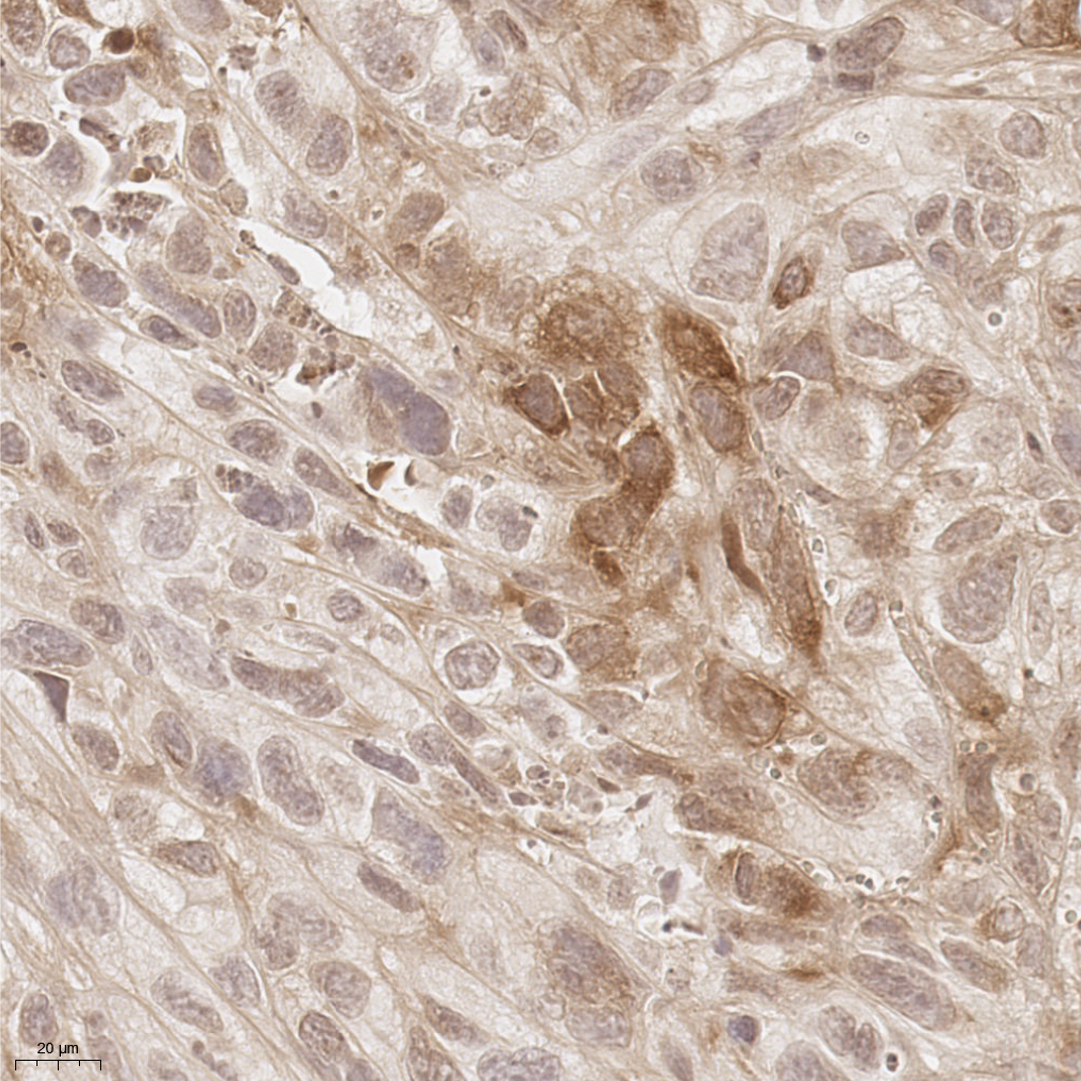

Supplement: Supplementary file 4 — Source data Fig. 2 [file 44321_2024_94_MOESM4_ESM.zip › Figure 2/2C Image Data micr. image/Bard1-def Res.tif]

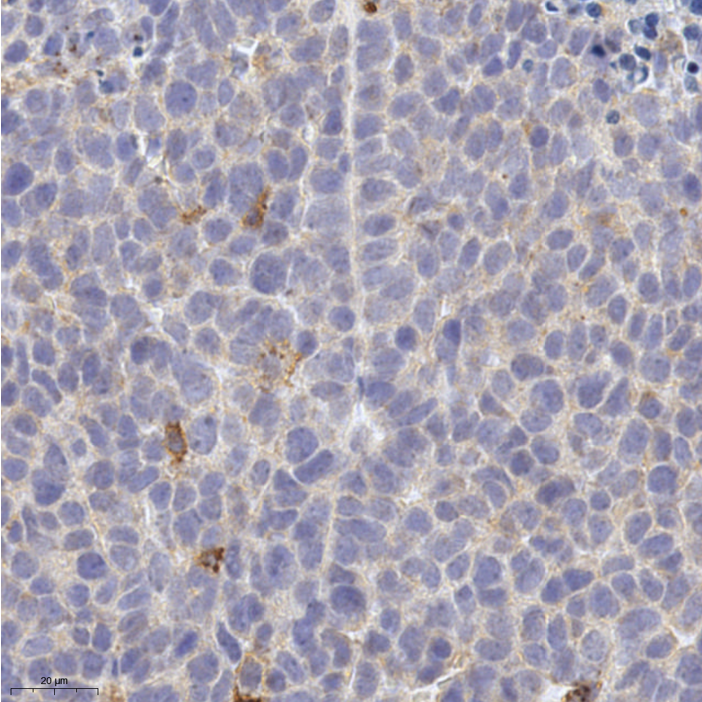

Supplement: Supplementary file 5 — Source data Fig. 3 [file 44321_2024_94_MOESM5_ESM.zip › Figure 3/3A Image Data micr. image/Brca1-def Sen.tif]

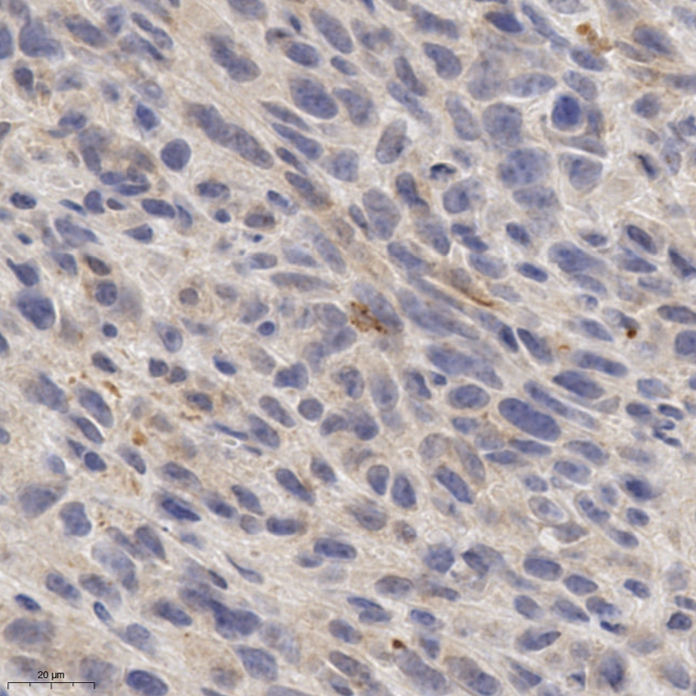

Supplement: Supplementary file 5 — Source data Fig. 3 [file 44321_2024_94_MOESM5_ESM.zip › Figure 3/3A Image Data micr. image/Bard1-def Sen.tif]

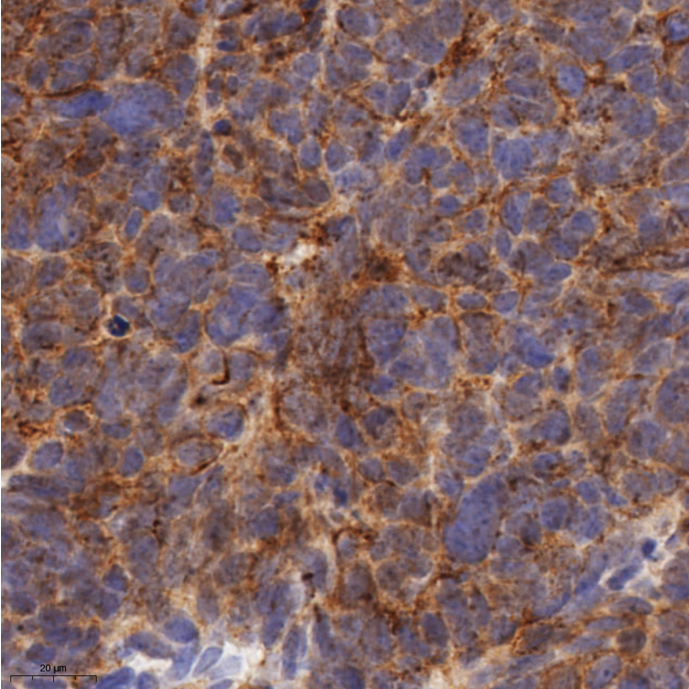

Supplement: Supplementary file 5 — Source data Fig. 3 [file 44321_2024_94_MOESM5_ESM.zip › Figure 3/3A Image Data micr. image/Brca1-def Res.tif]

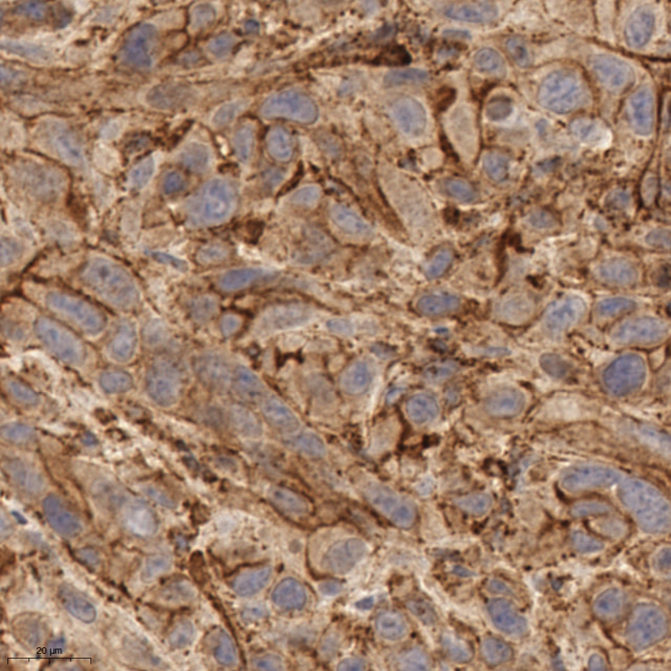

Supplement: Supplementary file 5 — Source data Fig. 3 [file 44321_2024_94_MOESM5_ESM.zip › Figure 3/3A Image Data micr. image/Bard1-def Res.tif]

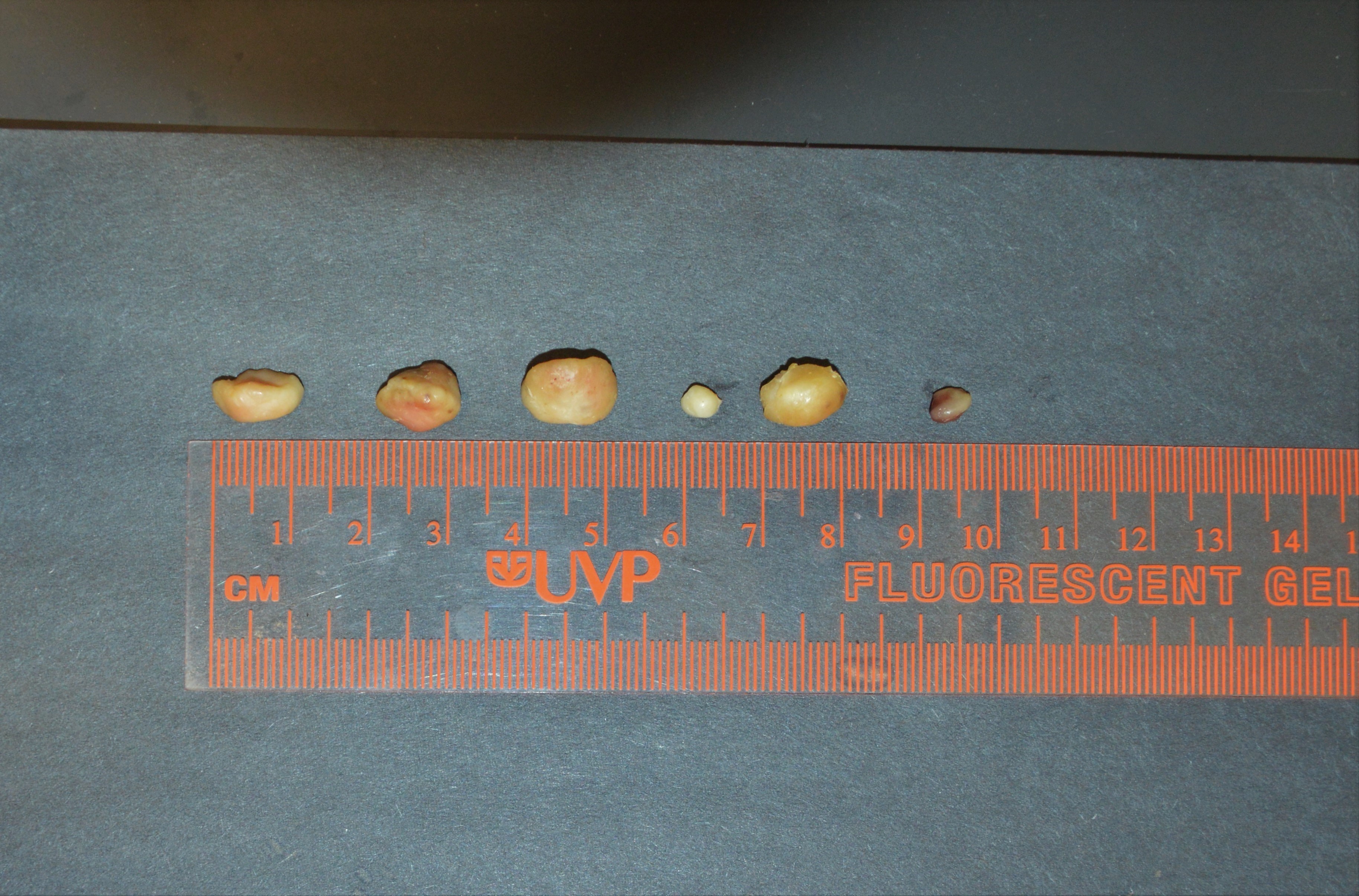

Supplement: Supplementary file 5 — Source data Fig. 3 [file 44321_2024_94_MOESM5_ESM.zip › Figure 3/3E Image Data Whole mount/Bard1 Tumor Image.tif]

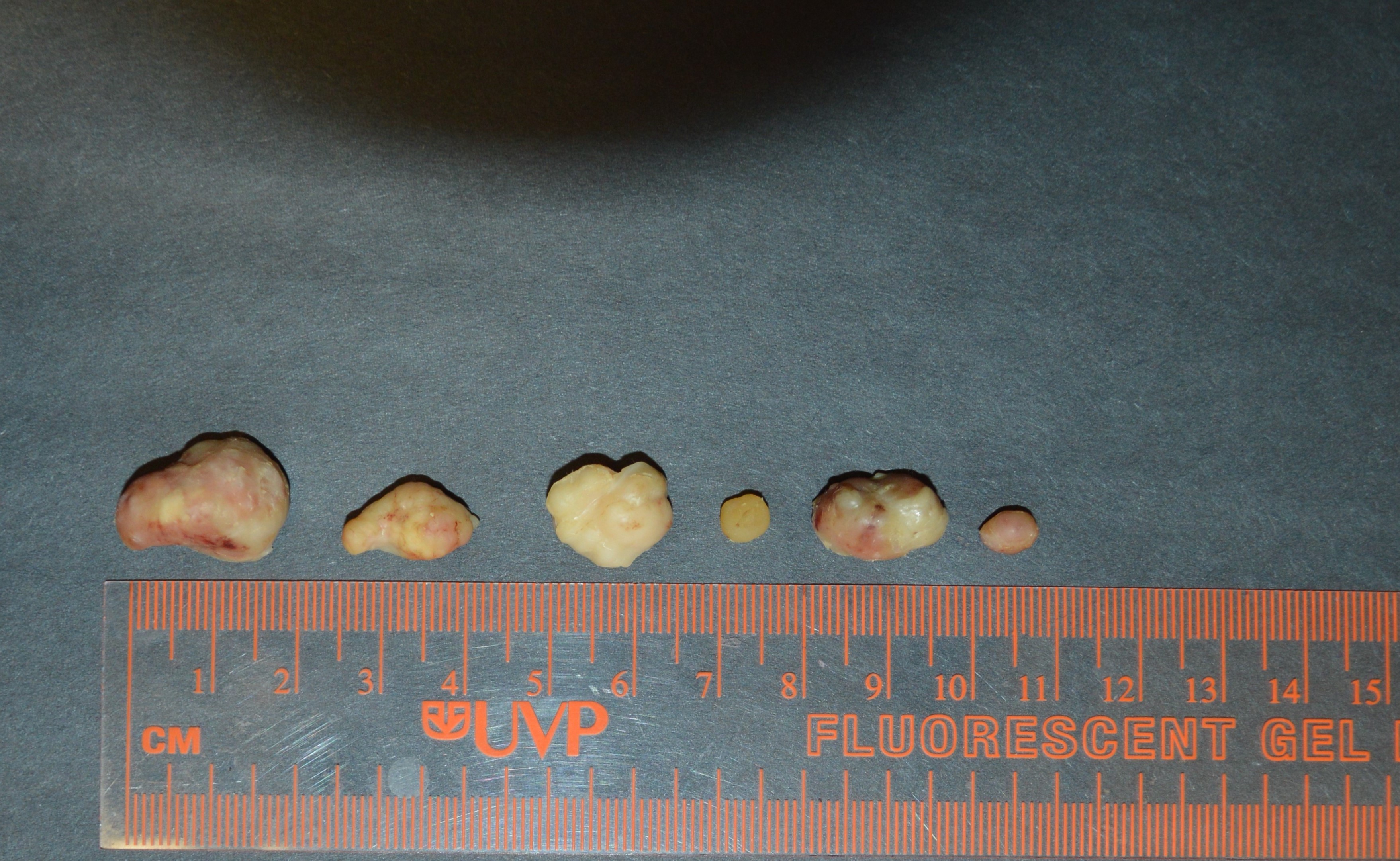

Supplement: Supplementary file 5 — Source data Fig. 3 [file 44321_2024_94_MOESM5_ESM.zip › Figure 3/3E Image Data Whole mount/Brca1 Tumor Image.tif]

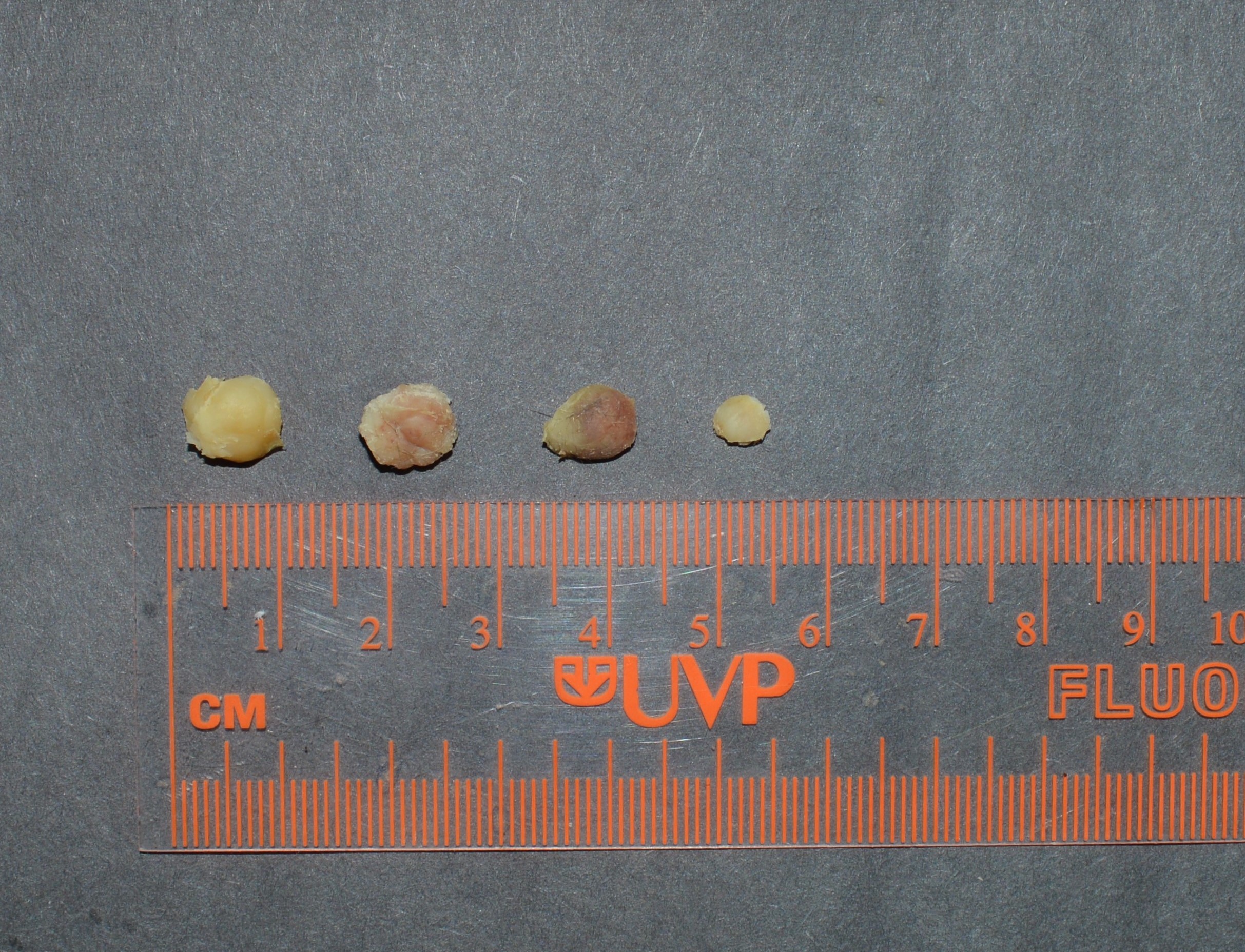

Supplement: Supplementary file 6 — Source data Fig. 4 [file 44321_2024_94_MOESM6_ESM.zip › Figure 4/4F Image data whole mount/Bard1-def Tumor Image.tif]

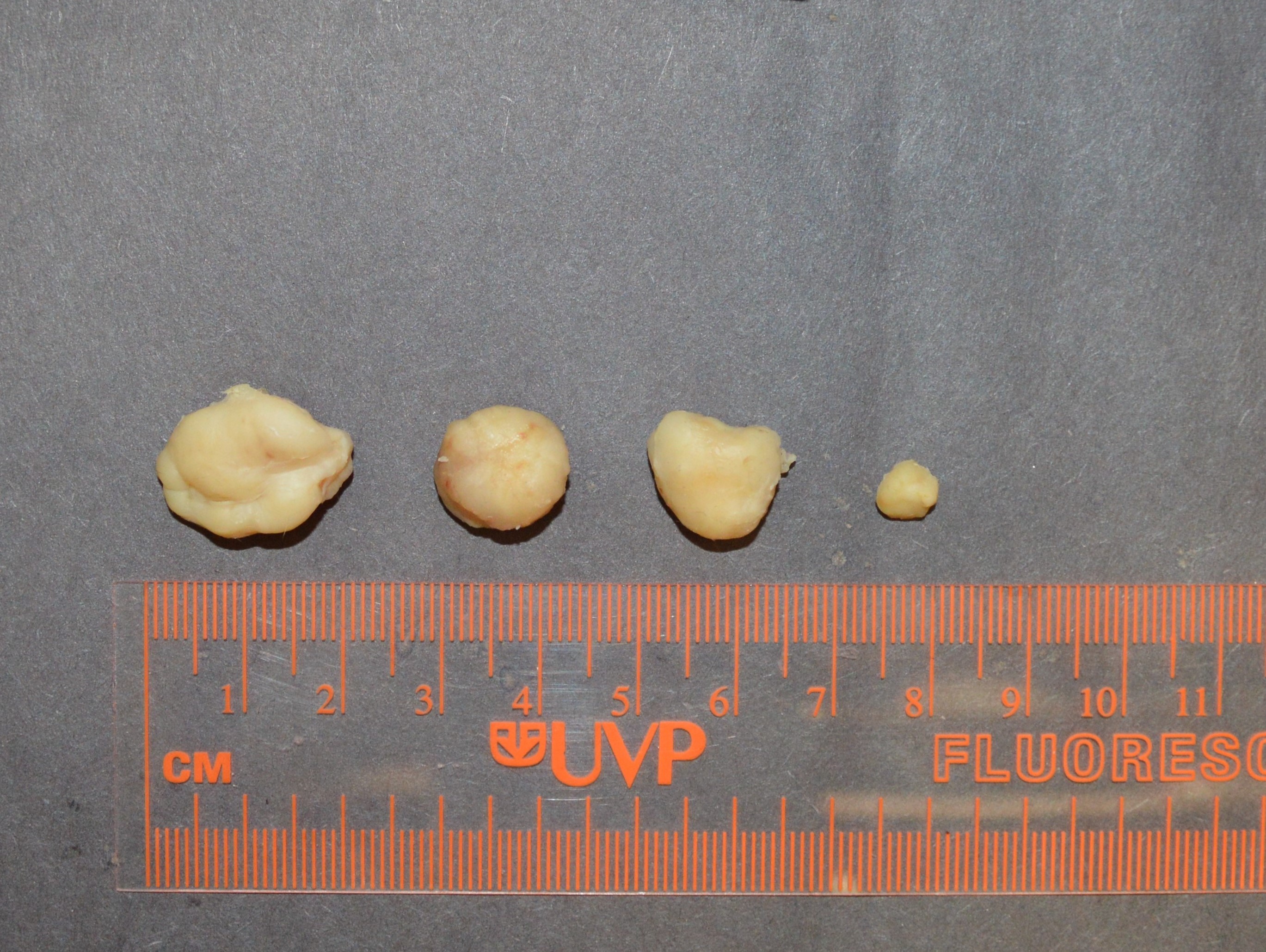

Supplement: Supplementary file 6 — Source data Fig. 4 [file 44321_2024_94_MOESM6_ESM.zip › Figure 4/4C Image data whole mount/Brca1-def Tumor Image.tif]

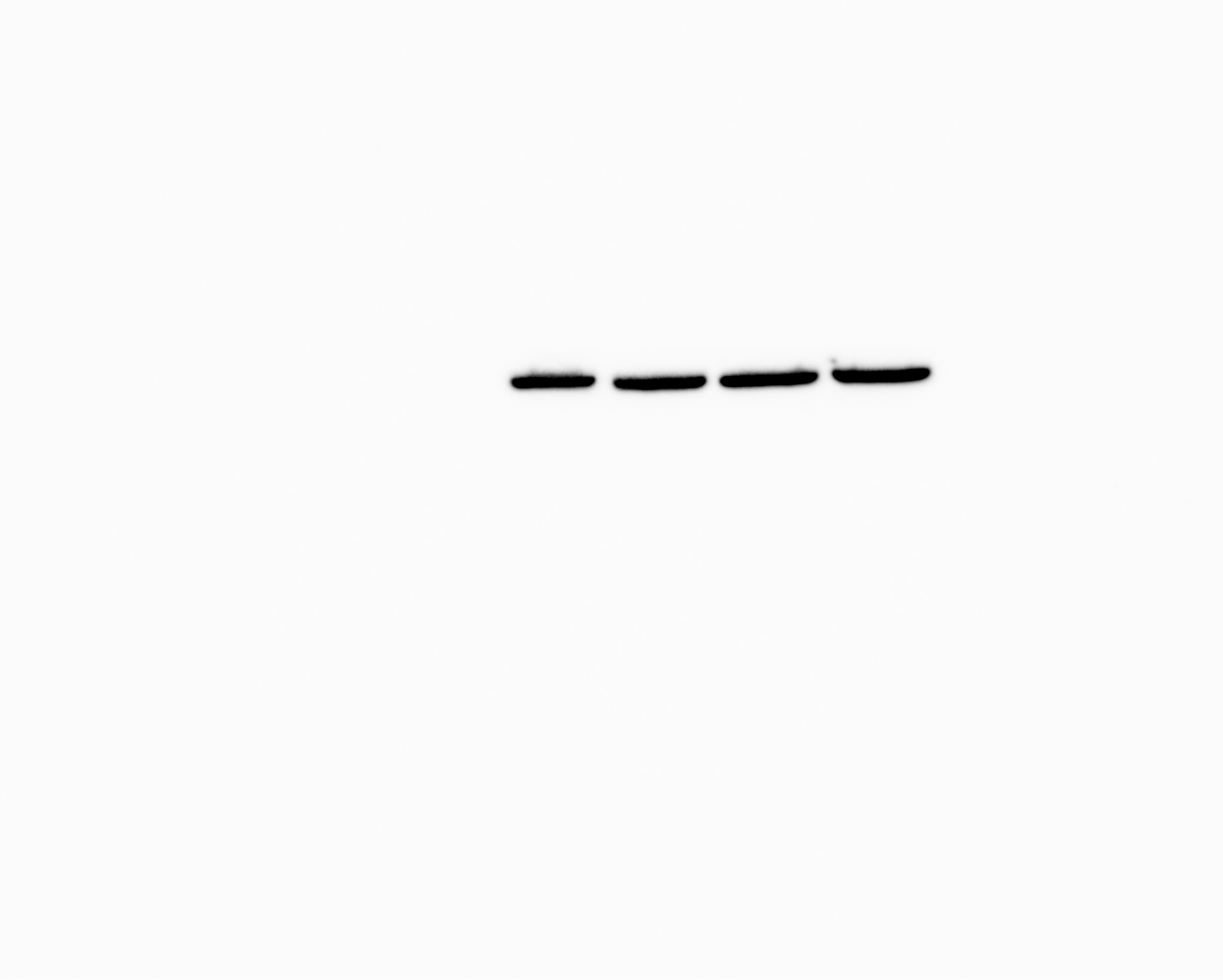

Supplement: Supplementary file 7 — Source data Fig. 5 [file 44321_2024_94_MOESM7_ESM.zip › Figure 5/5B Image data blot/┬╖-actin.tif]

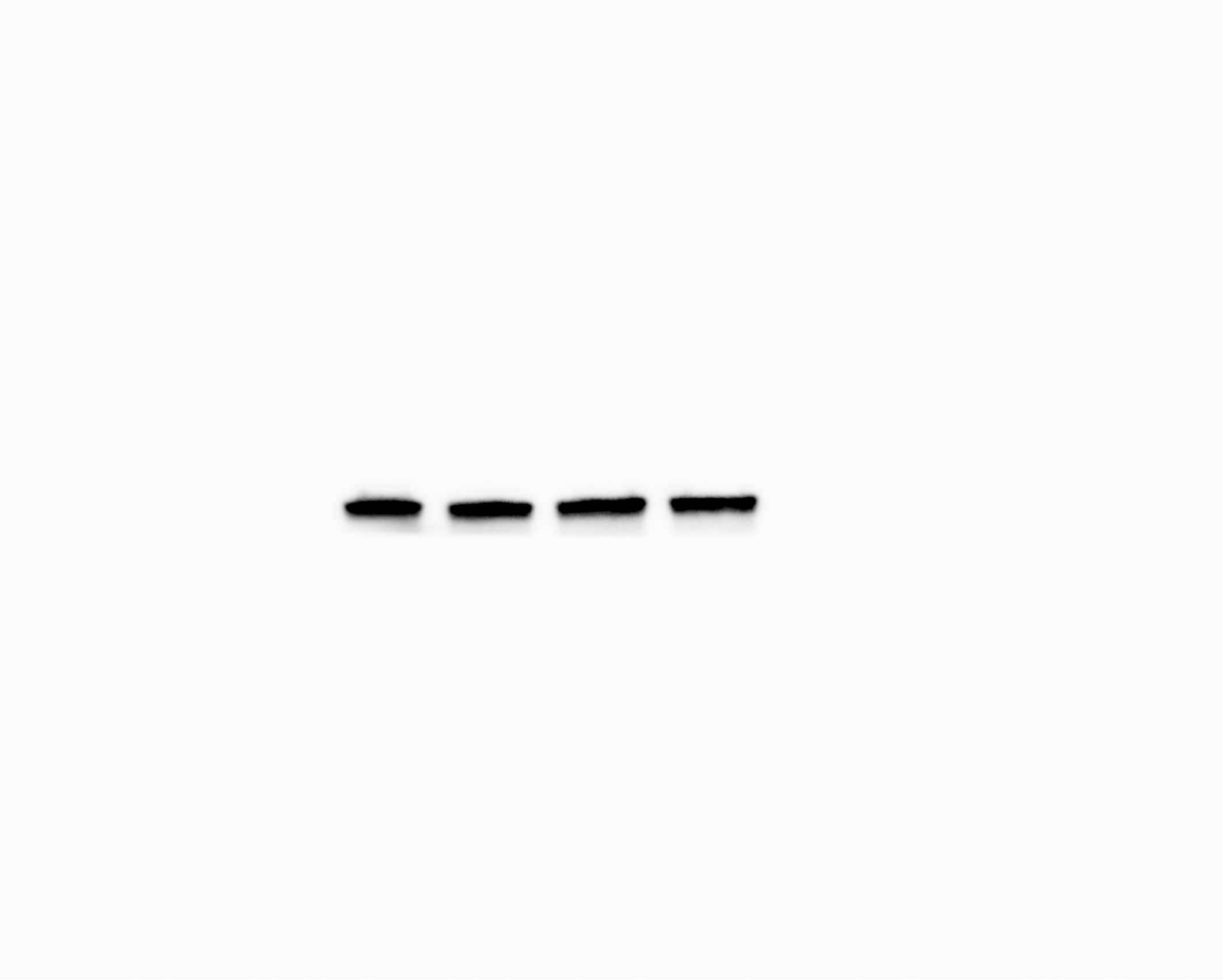

Supplement: Supplementary file 7 — Source data Fig. 5 [file 44321_2024_94_MOESM7_ESM.zip › Figure 5/5B Image data blot/AKT.tif]

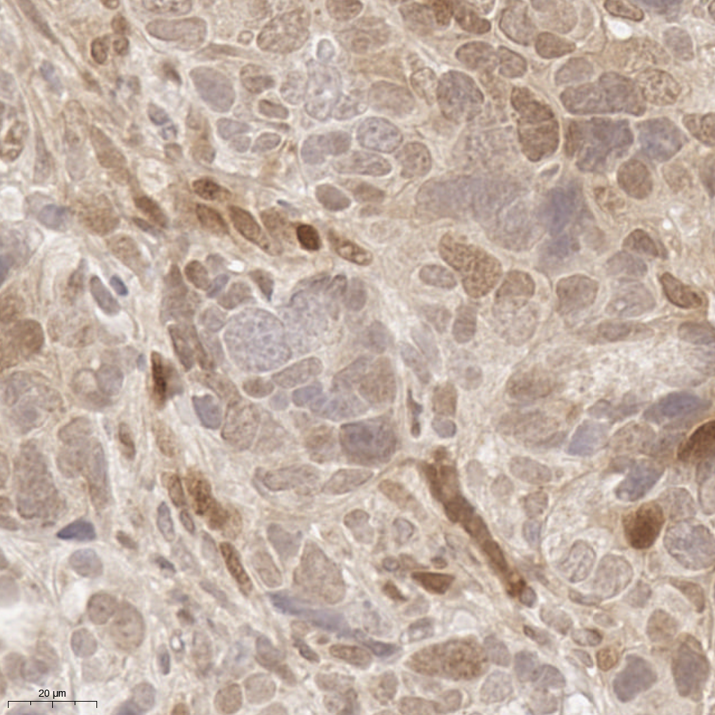

Supplement: Supplementary file 7 — Source data Fig. 5 [file 44321_2024_94_MOESM7_ESM.zip › Figure 5/5G Image data micr. image/Brca1-def Tal + Axi.tif]

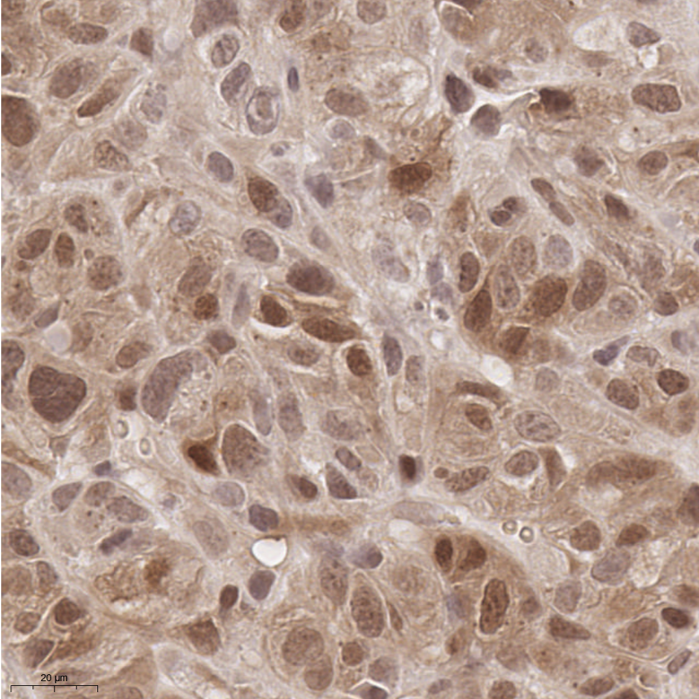

Supplement: Supplementary file 7 — Source data Fig. 5 [file 44321_2024_94_MOESM7_ESM.zip › Figure 5/5G Image data micr. image/Bard1-def Tal.tif]

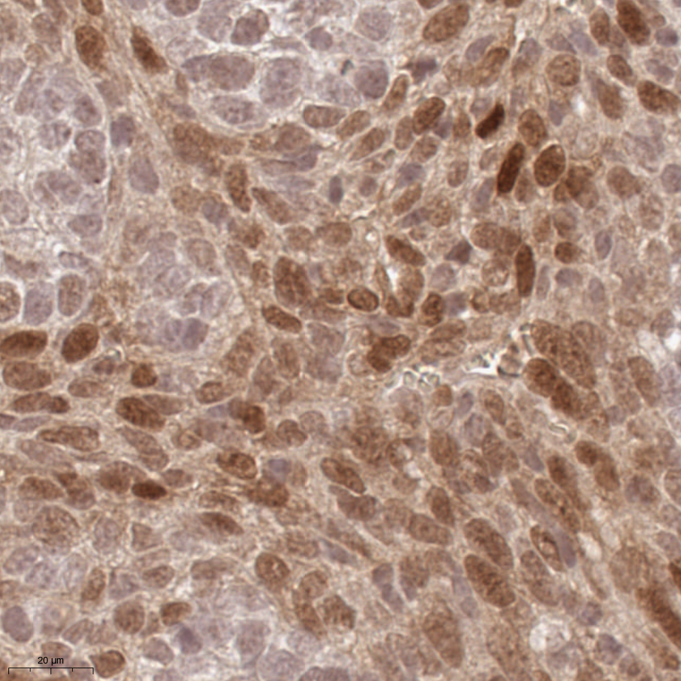

Supplement: Supplementary file 7 — Source data Fig. 5 [file 44321_2024_94_MOESM7_ESM.zip › Figure 5/5G Image data micr. image/Brca1-def Tal.tif]

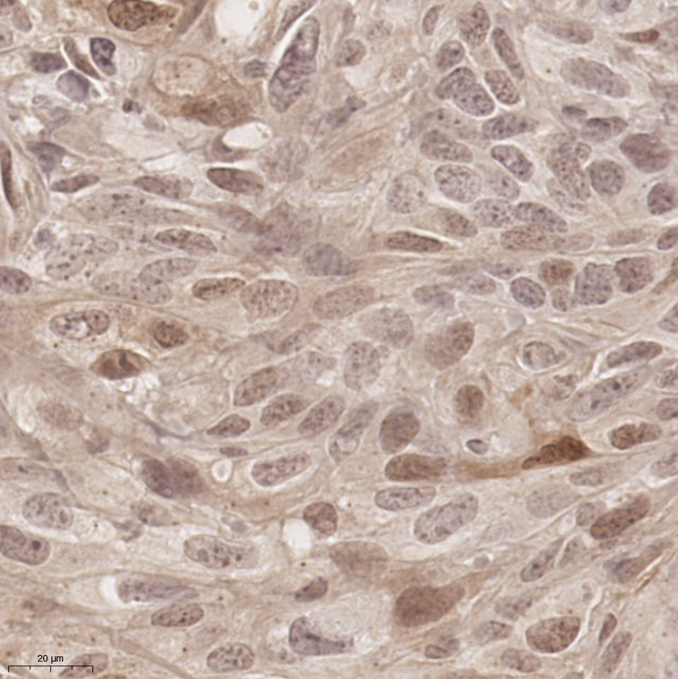

Supplement: Supplementary file 7 — Source data Fig. 5 [file 44321_2024_94_MOESM7_ESM.zip › Figure 5/5G Image data micr. image/Bard1-def Tal + Axi.tif]

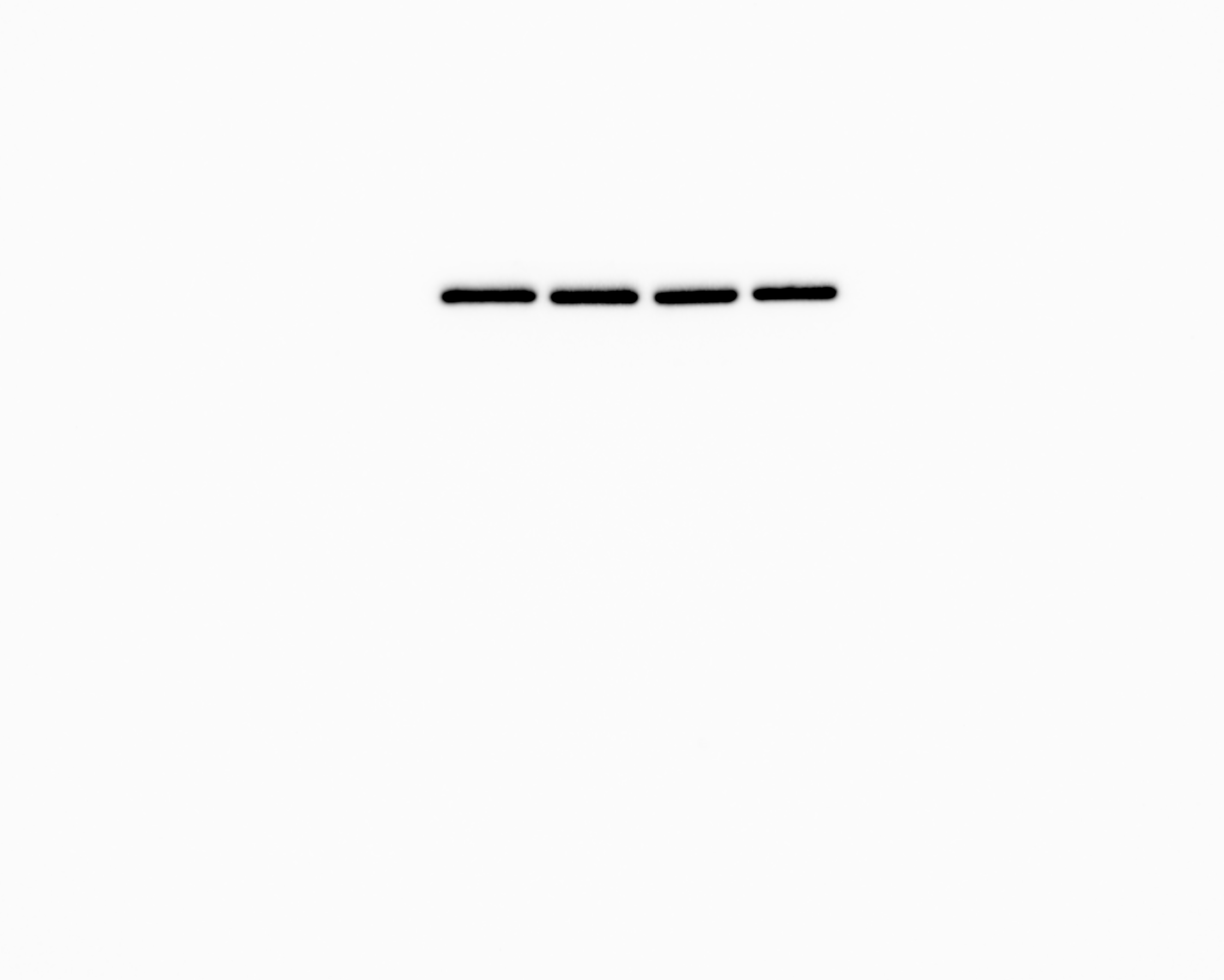

Supplement: Supplementary file 7 — Source data Fig. 5 [file 44321_2024_94_MOESM7_ESM.zip › Figure 5/5A Image Data blot/┬╖-actin.tif]

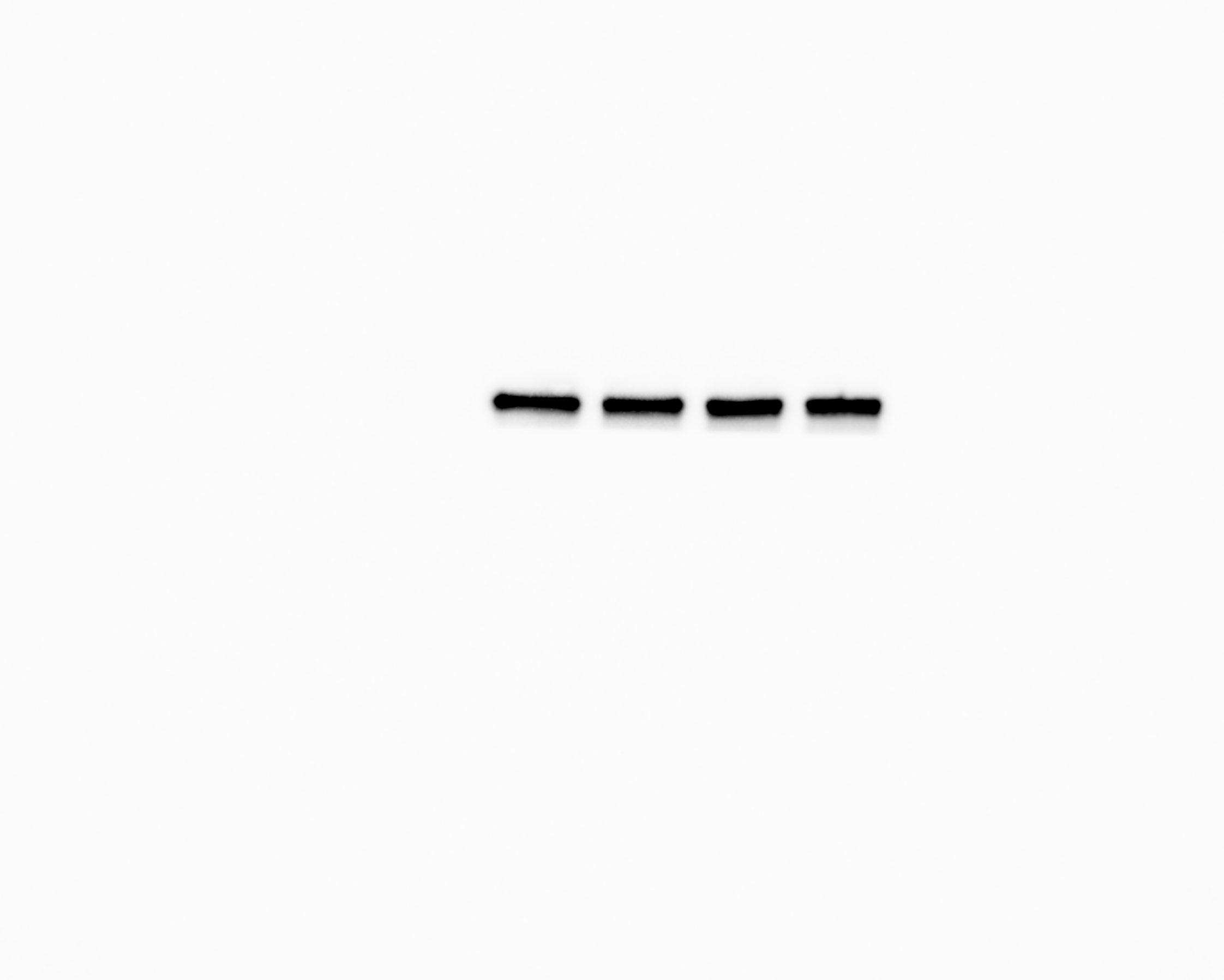

Supplement: Supplementary file 7 — Source data Fig. 5 [file 44321_2024_94_MOESM7_ESM.zip › Figure 5/5A Image Data blot/AKT.tif]

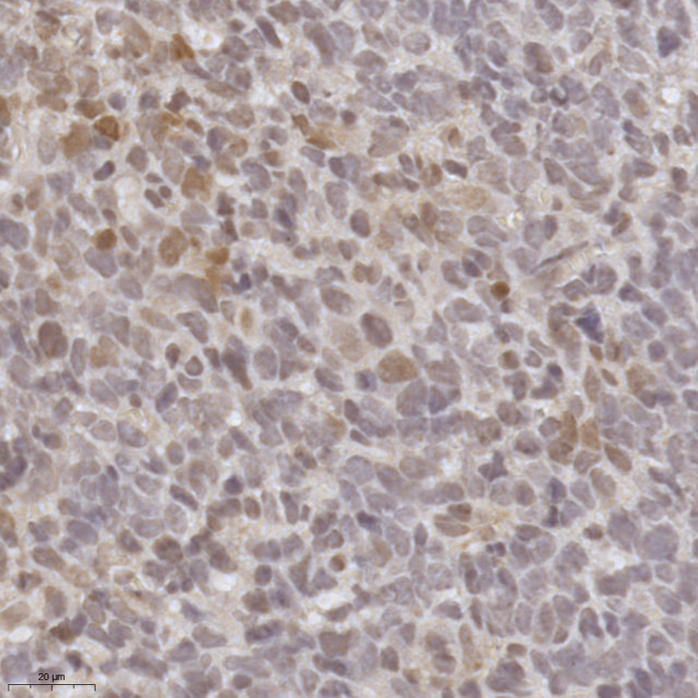

Supplement: Supplementary file 7 — Source data Fig. 5 [file 44321_2024_94_MOESM7_ESM.zip › Figure 5/5C Image Data Micr. image/Brca1-def Sen.tif]

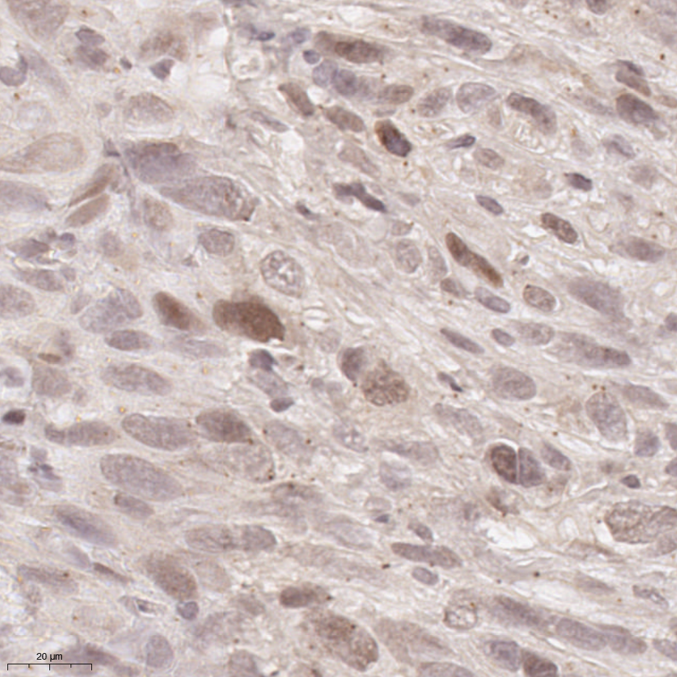

Supplement: Supplementary file 7 — Source data Fig. 5 [file 44321_2024_94_MOESM7_ESM.zip › Figure 5/5C Image Data Micr. image/Bard1-def Sen.tif]

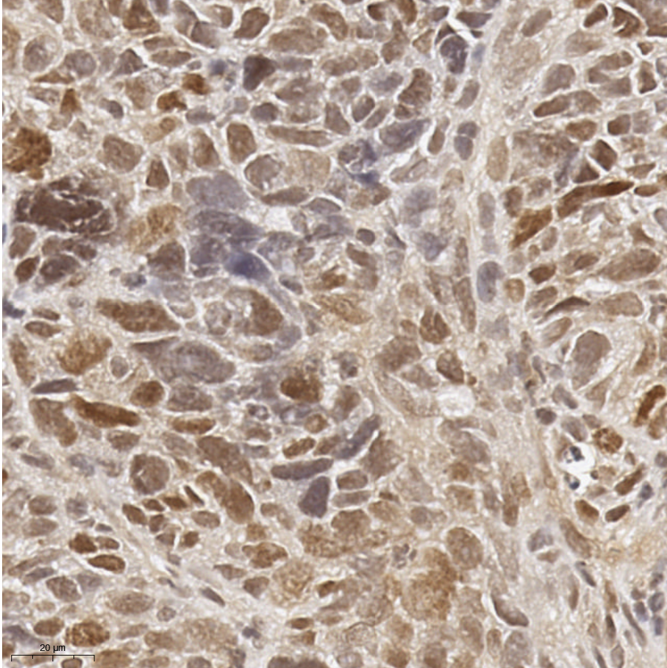

Supplement: Supplementary file 7 — Source data Fig. 5 [file 44321_2024_94_MOESM7_ESM.zip › Figure 5/5C Image Data Micr. image/Brca1-def Res.tif]

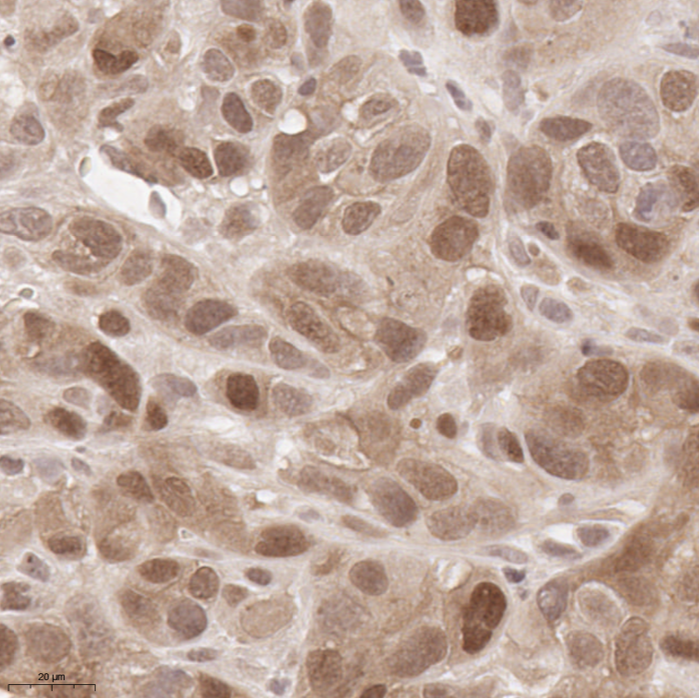

Supplement: Supplementary file 7 — Source data Fig. 5 [file 44321_2024_94_MOESM7_ESM.zip › Figure 5/5C Image Data Micr. image/Bard1-def Res.tif]

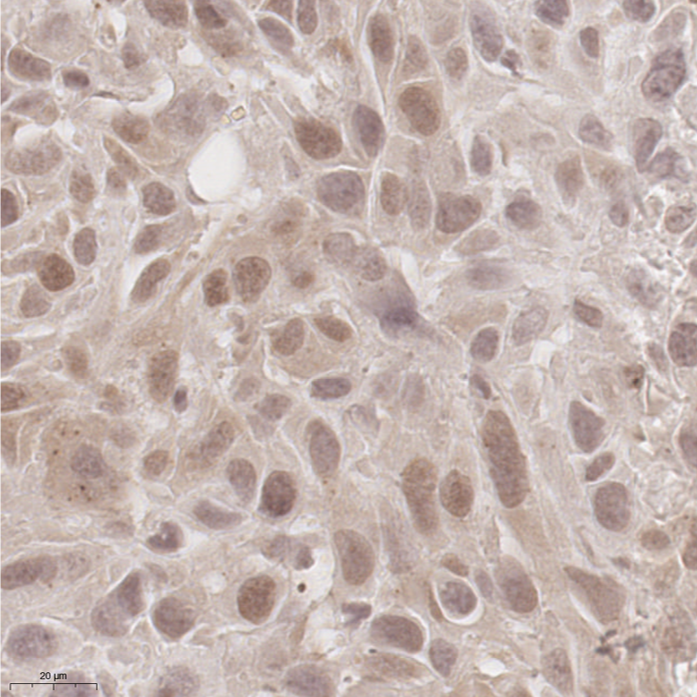

Supplement: Supplementary file 7 — Source data Fig. 5 [file 44321_2024_94_MOESM7_ESM.zip › Figure 5/5E Image data Micr. image/Bard1-def Flt1i +Tal.tif]

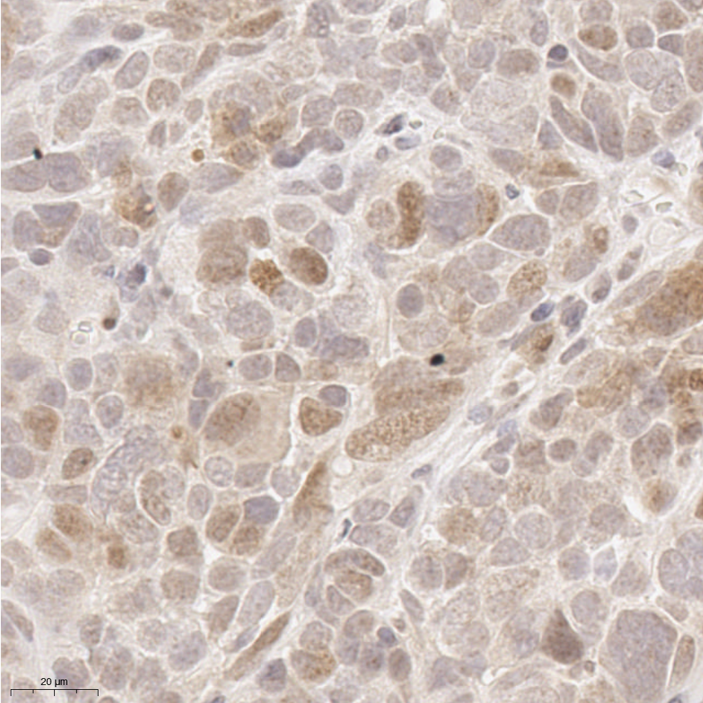

Supplement: Supplementary file 7 — Source data Fig. 5 [file 44321_2024_94_MOESM7_ESM.zip › Figure 5/5E Image data Micr. image/Brca1-def Flt1i +Tal.tif]

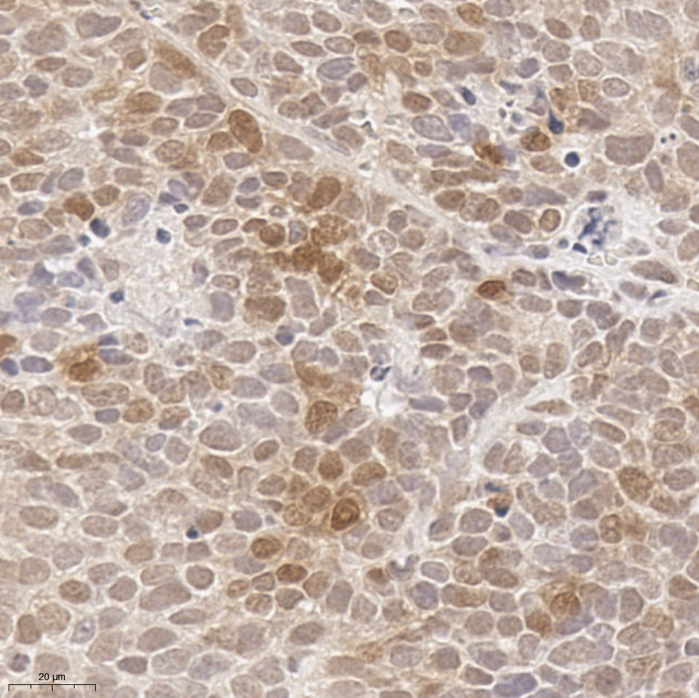

Supplement: Supplementary file 7 — Source data Fig. 5 [file 44321_2024_94_MOESM7_ESM.zip › Figure 5/5E Image data Micr. image/Brca1-def Lenti-Con+Tal.tif]

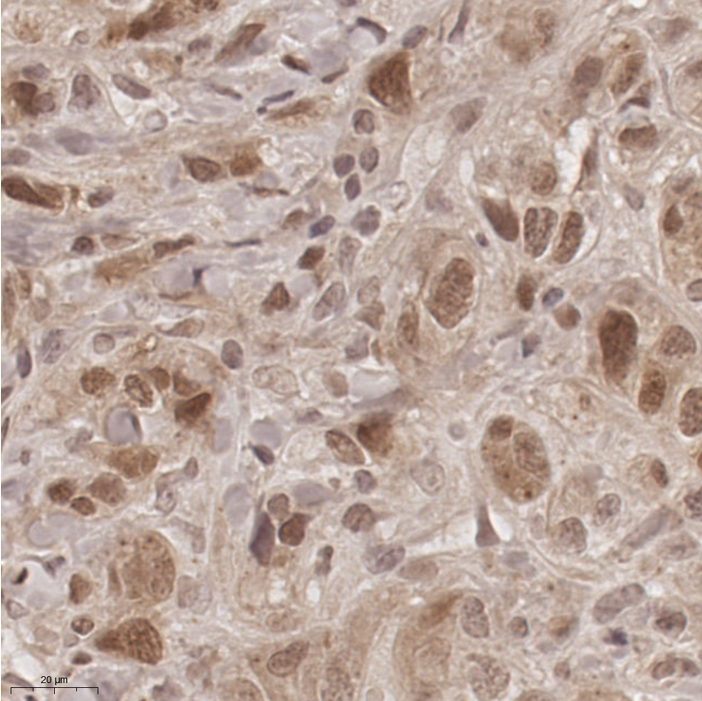

Supplement: Supplementary file 7 — Source data Fig. 5 [file 44321_2024_94_MOESM7_ESM.zip › Figure 5/5E Image data Micr. image/Bard1-def Lenti-Con+Tal.tif]

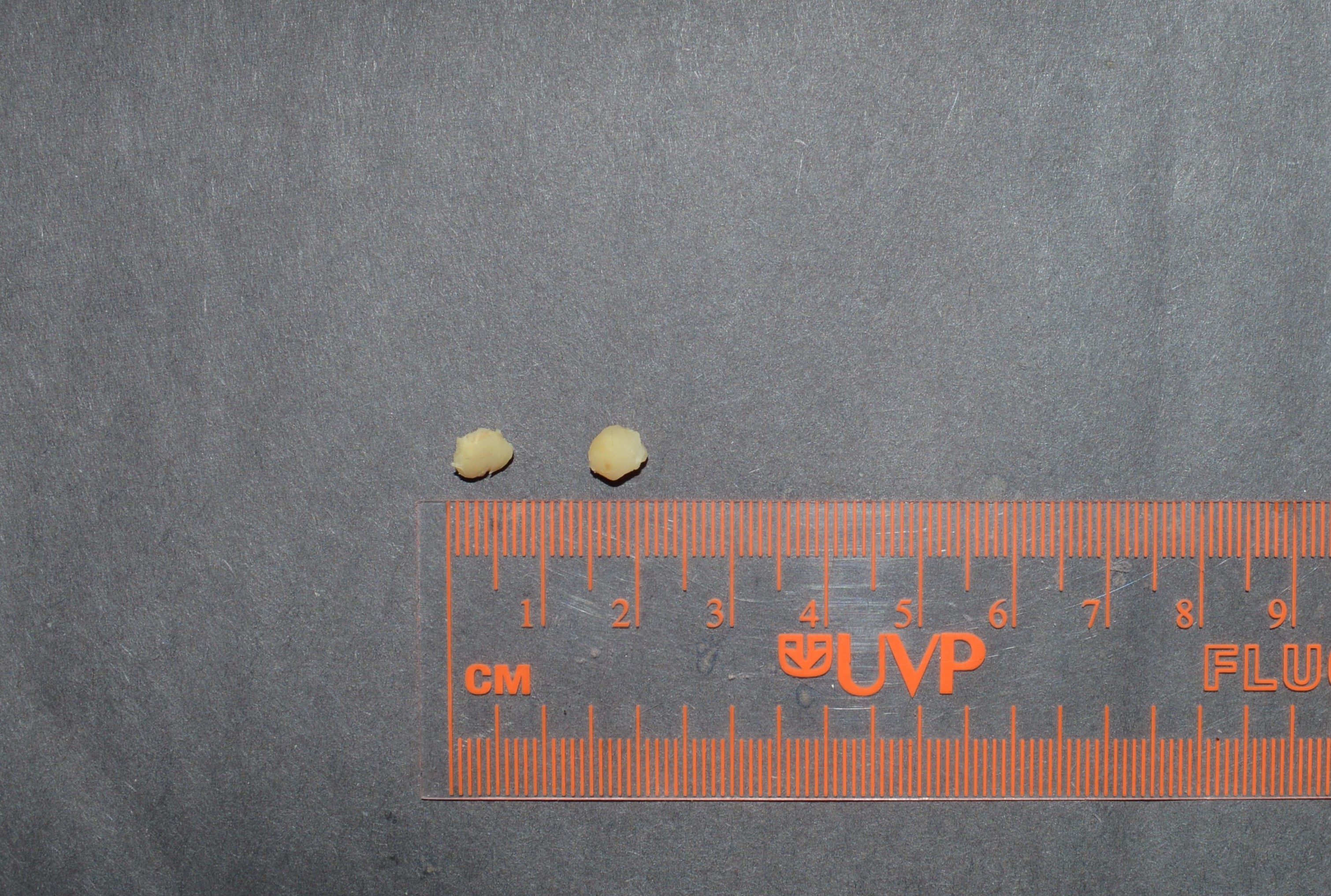

Supplement: Supplementary file 8 — Source data Fig. 6 [file 44321_2024_94_MOESM8_ESM.zip › Figure 6/6I Image data whole mount/Bard1-def Tumor Image.tif]

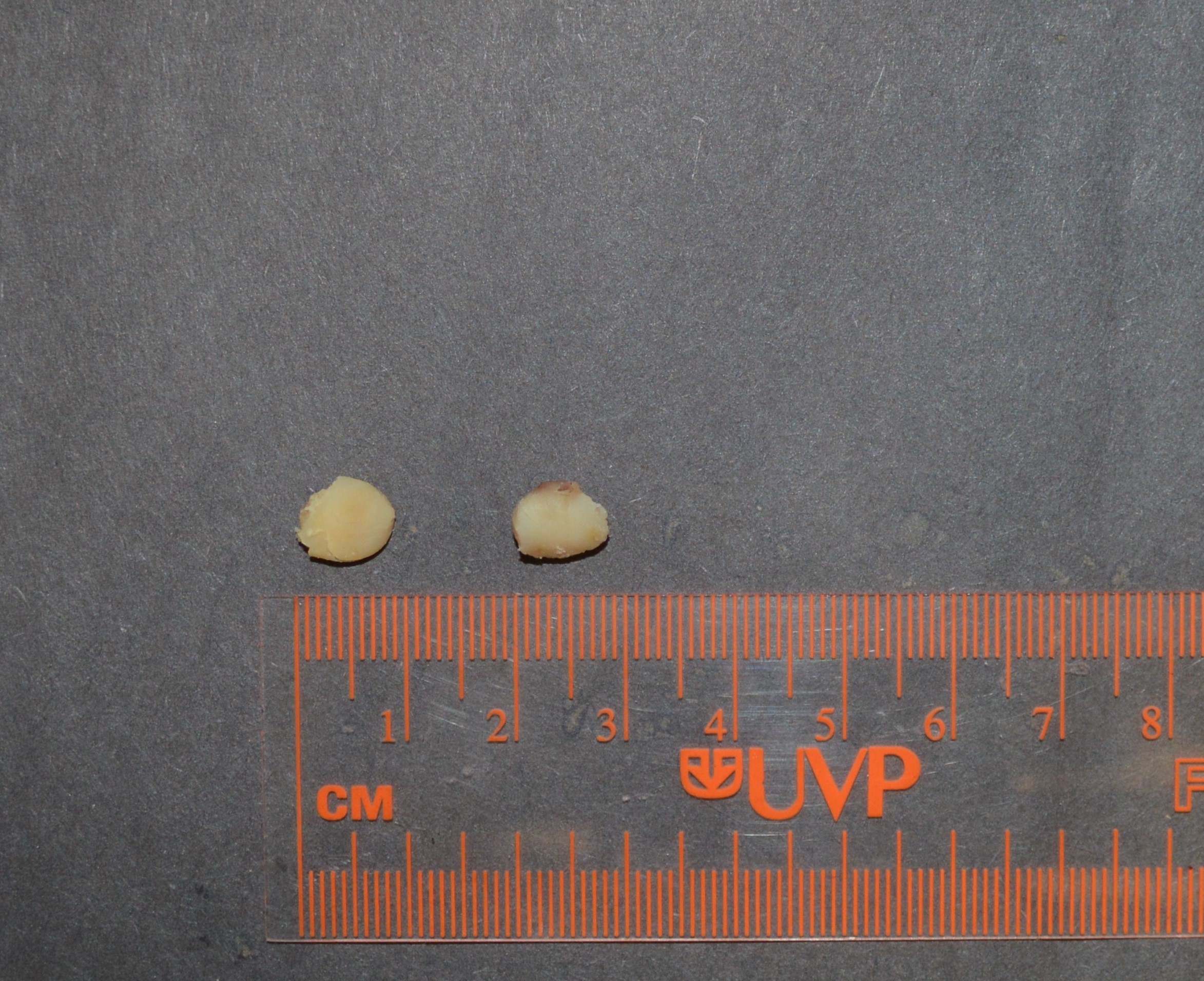

Supplement: Supplementary file 8 — Source data Fig. 6 [file 44321_2024_94_MOESM8_ESM.zip › Figure 6/6I Image data whole mount/Brca1-def Tumor Image.tif]

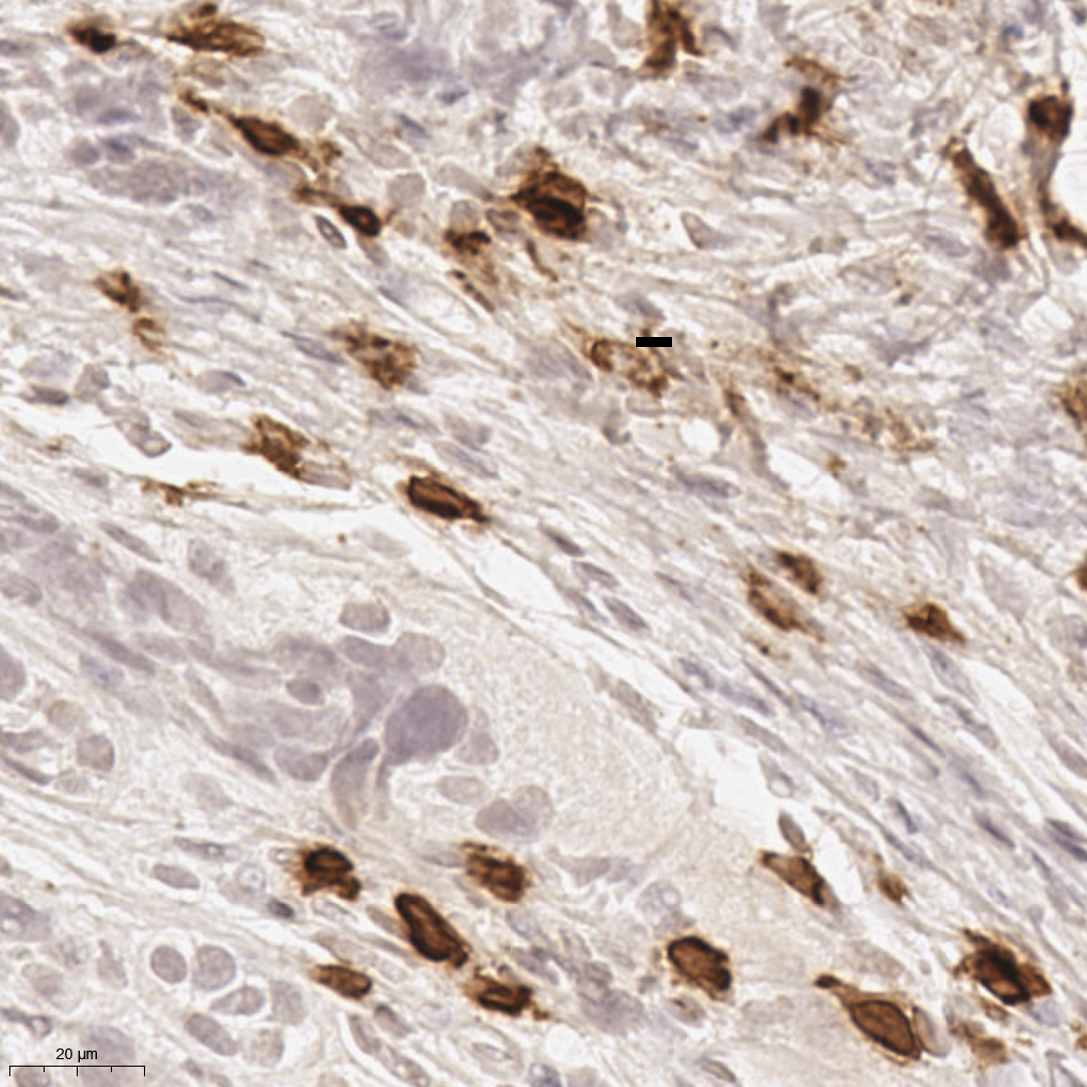

Supplement: Supplementary file 8 — Source data Fig. 6 [file 44321_2024_94_MOESM8_ESM.zip › Figure 6/6F Image data micr. image/Brca1-def Tal + Axi.tif]

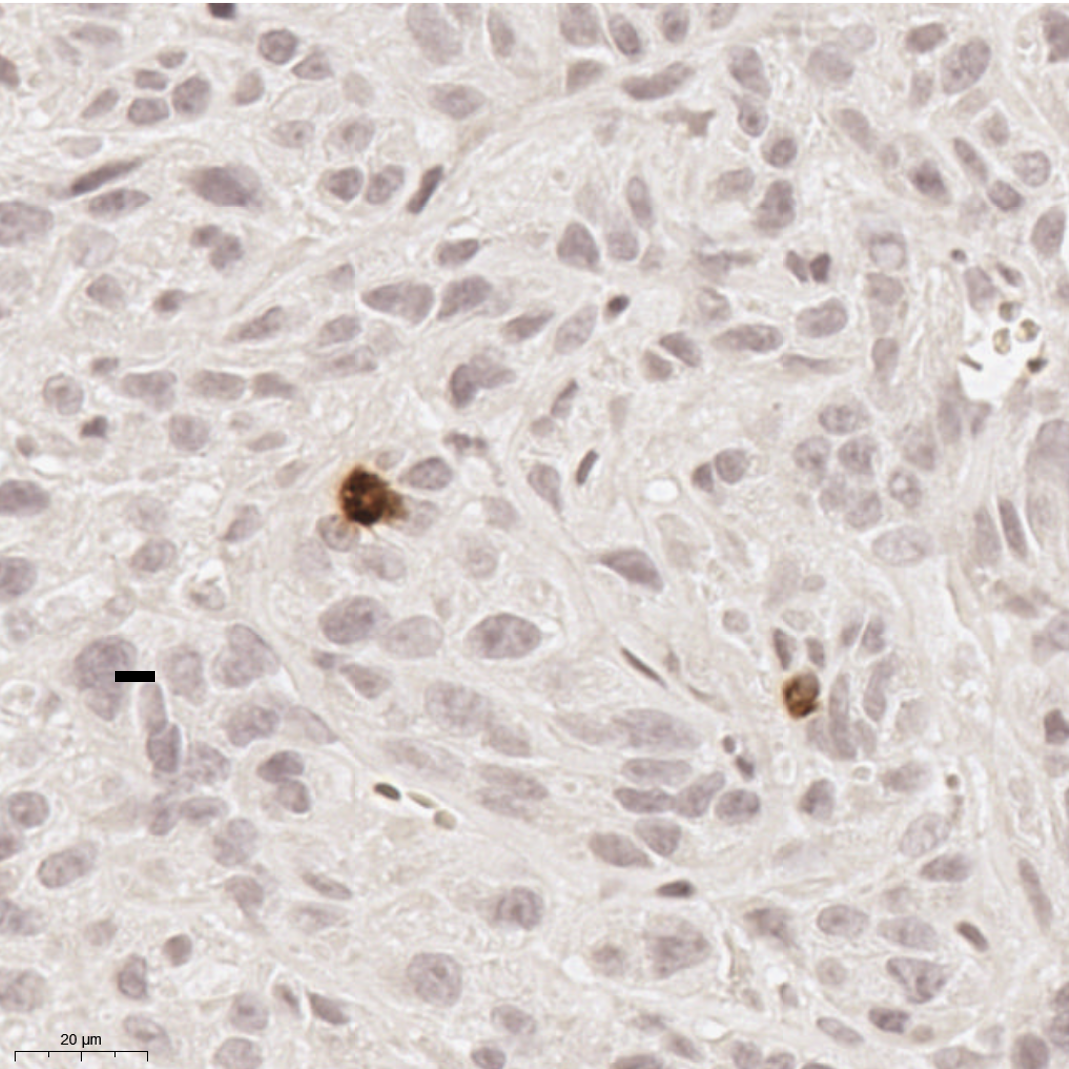

Supplement: Supplementary file 8 — Source data Fig. 6 [file 44321_2024_94_MOESM8_ESM.zip › Figure 6/6F Image data micr. image/Bard1-def Tal.tif]

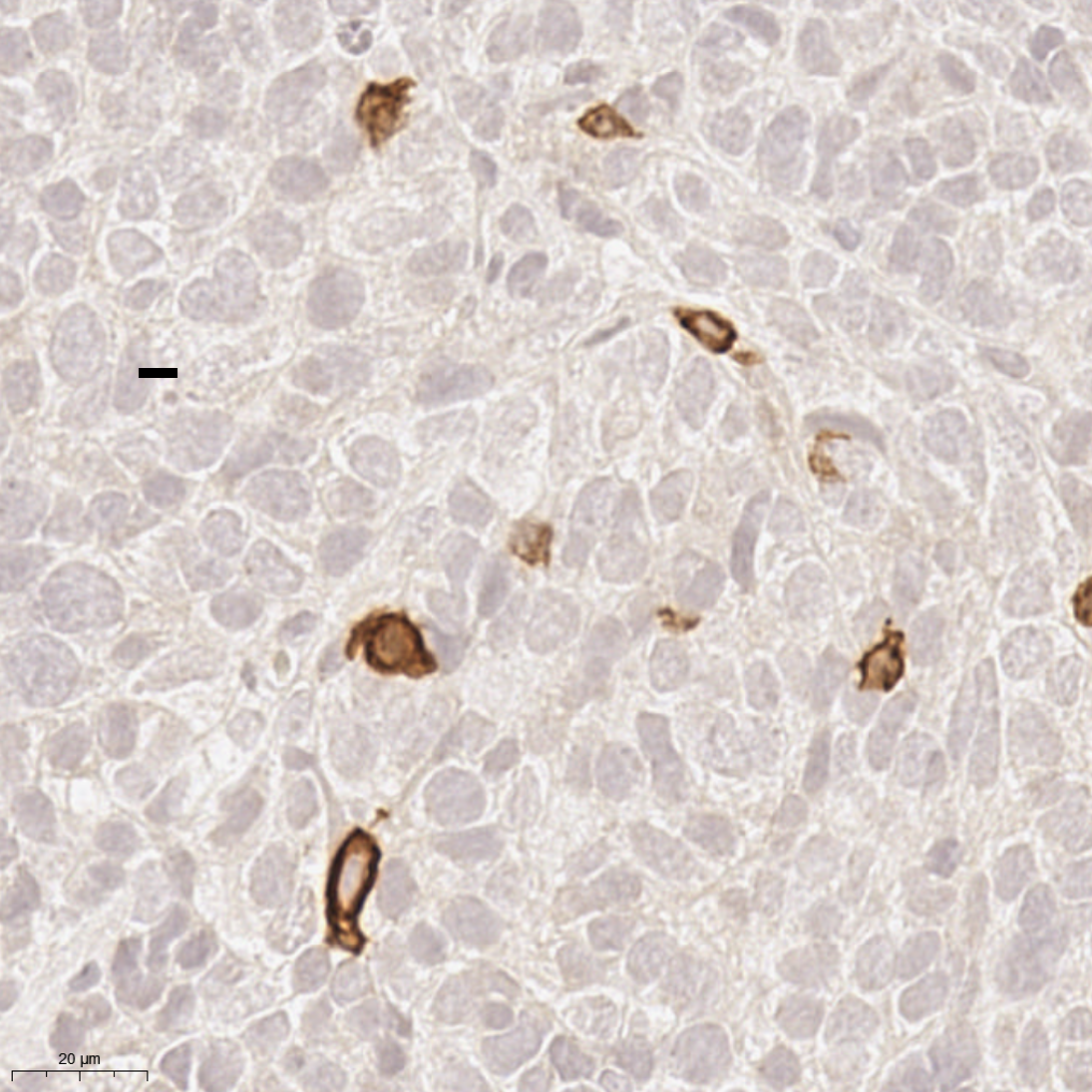

Supplement: Supplementary file 8 — Source data Fig. 6 [file 44321_2024_94_MOESM8_ESM.zip › Figure 6/6F Image data micr. image/Brca1-def Tal.tif]

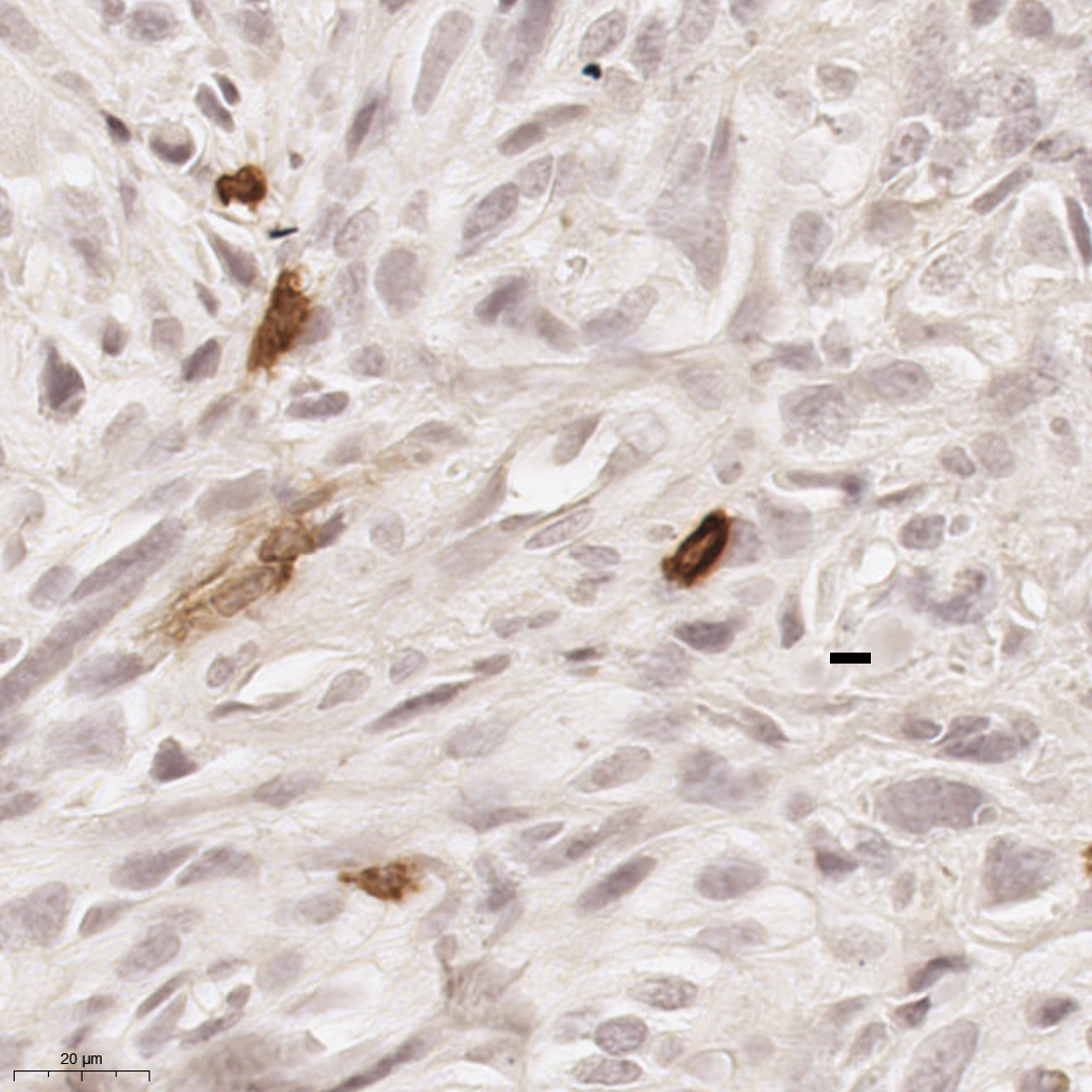

Supplement: Supplementary file 8 — Source data Fig. 6 [file 44321_2024_94_MOESM8_ESM.zip › Figure 6/6F Image data micr. image/Bard1-def Tal + Axi.tif]

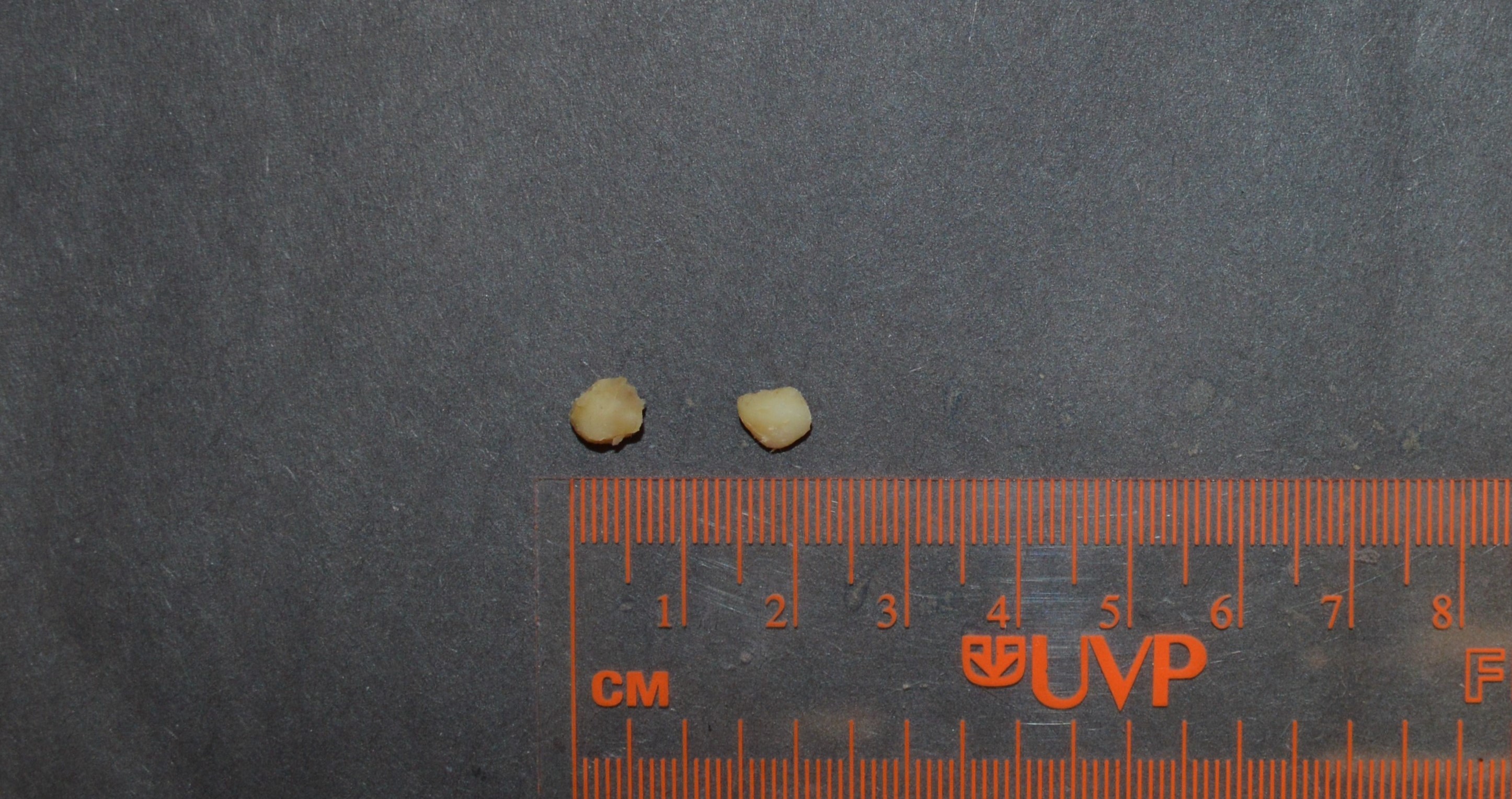

Supplement: Supplementary file 8 — Source data Fig. 6 [file 44321_2024_94_MOESM8_ESM.zip › Figure 6/6D Image data whole mount/Bard1-def Tumor Image.tif]

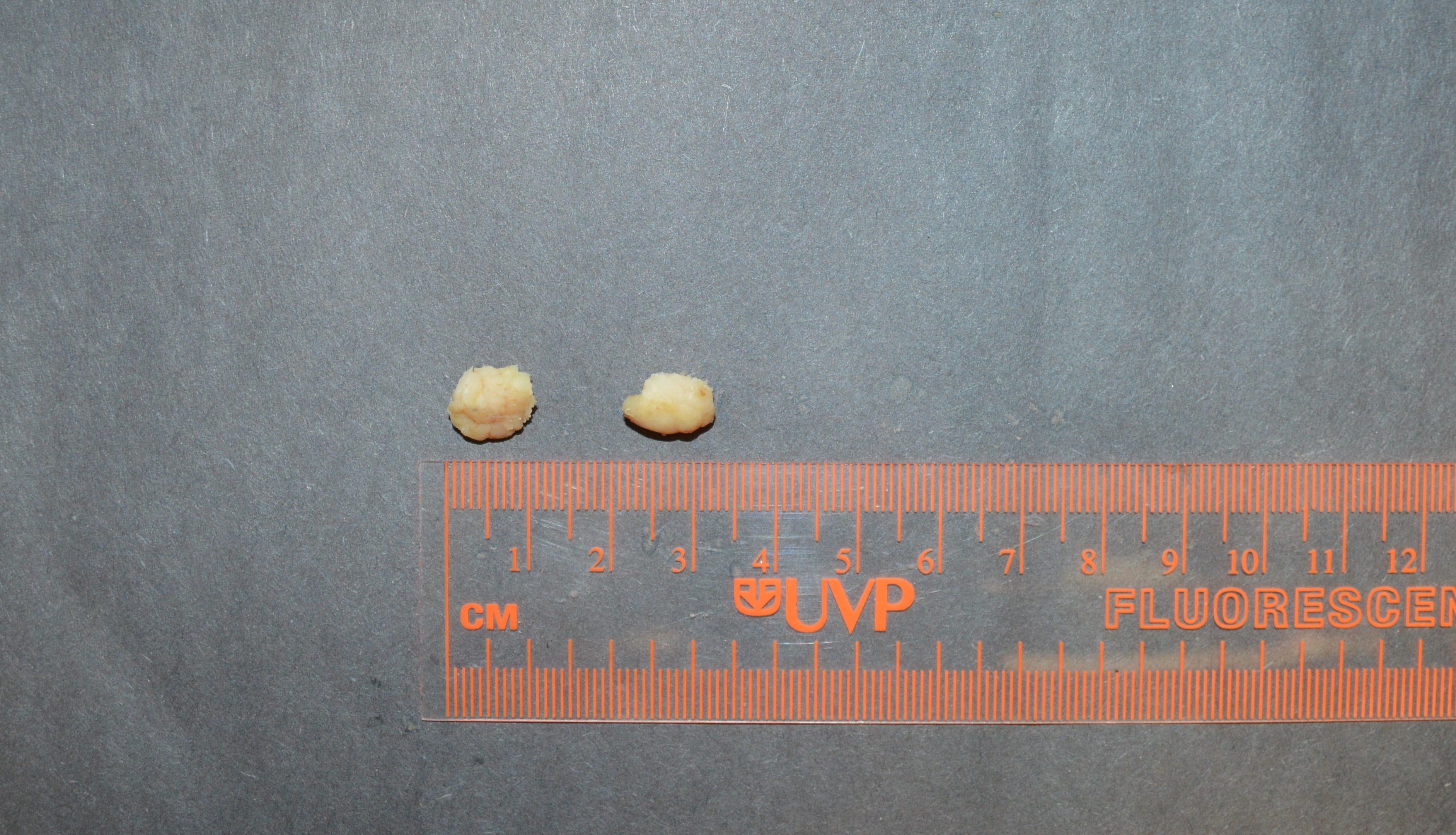

Supplement: Supplementary file 8 — Source data Fig. 6 [file 44321_2024_94_MOESM8_ESM.zip › Figure 6/6D Image data whole mount/Brca1-def Tumor Image.tif]

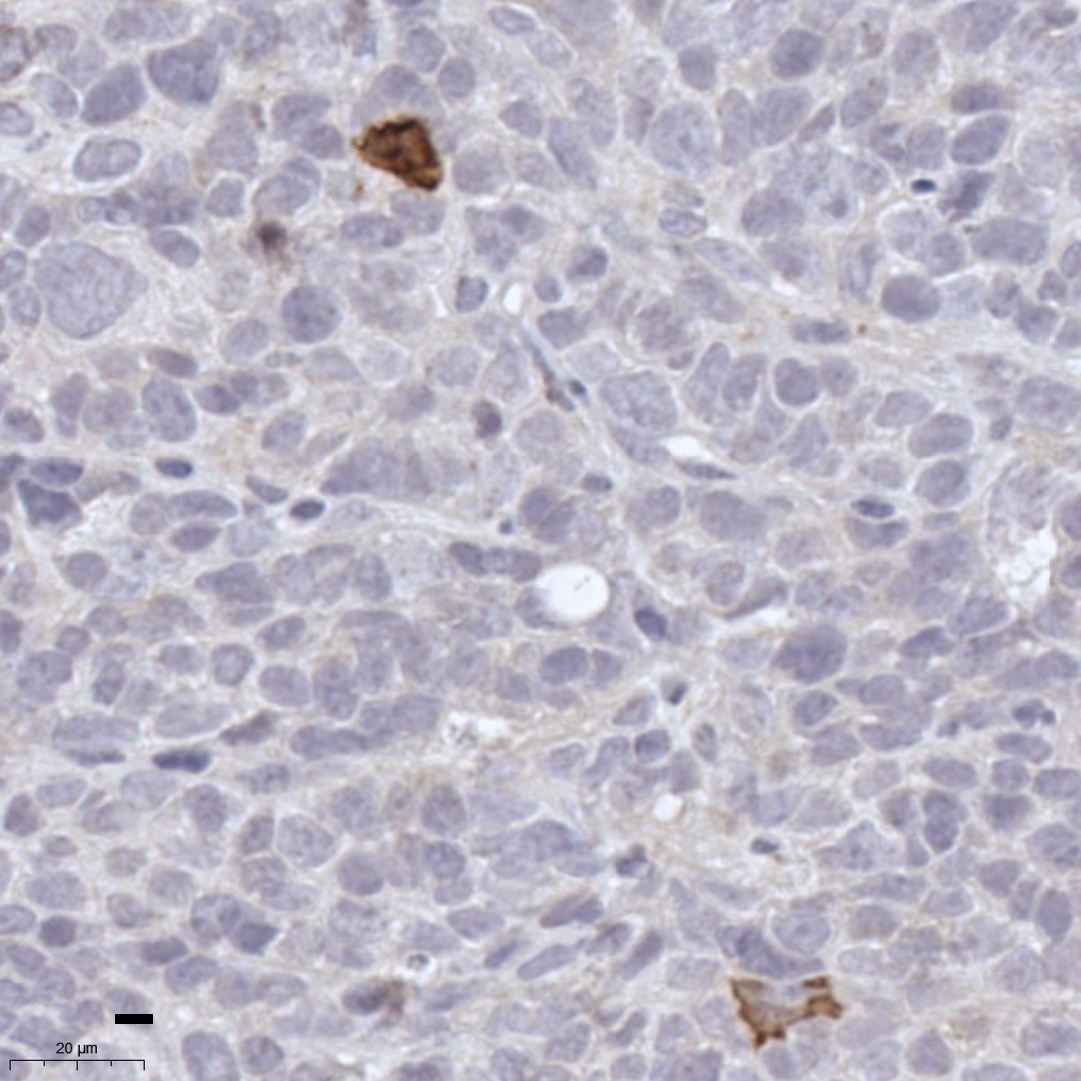

Supplement: Supplementary file 8 — Source data Fig. 6 [file 44321_2024_94_MOESM8_ESM.zip › Figure 6/6A Image data micr. image/Brca1def Lenti-Con + Tal.tif]

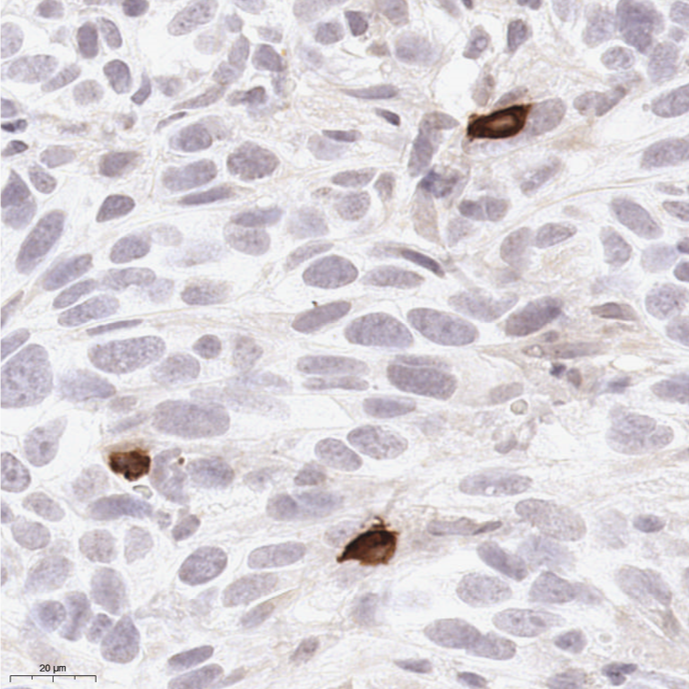

Supplement: Supplementary file 8 — Source data Fig. 6 [file 44321_2024_94_MOESM8_ESM.zip › Figure 6/6A Image data micr. image/Bard1def Lenti-Con + Tal.tif]

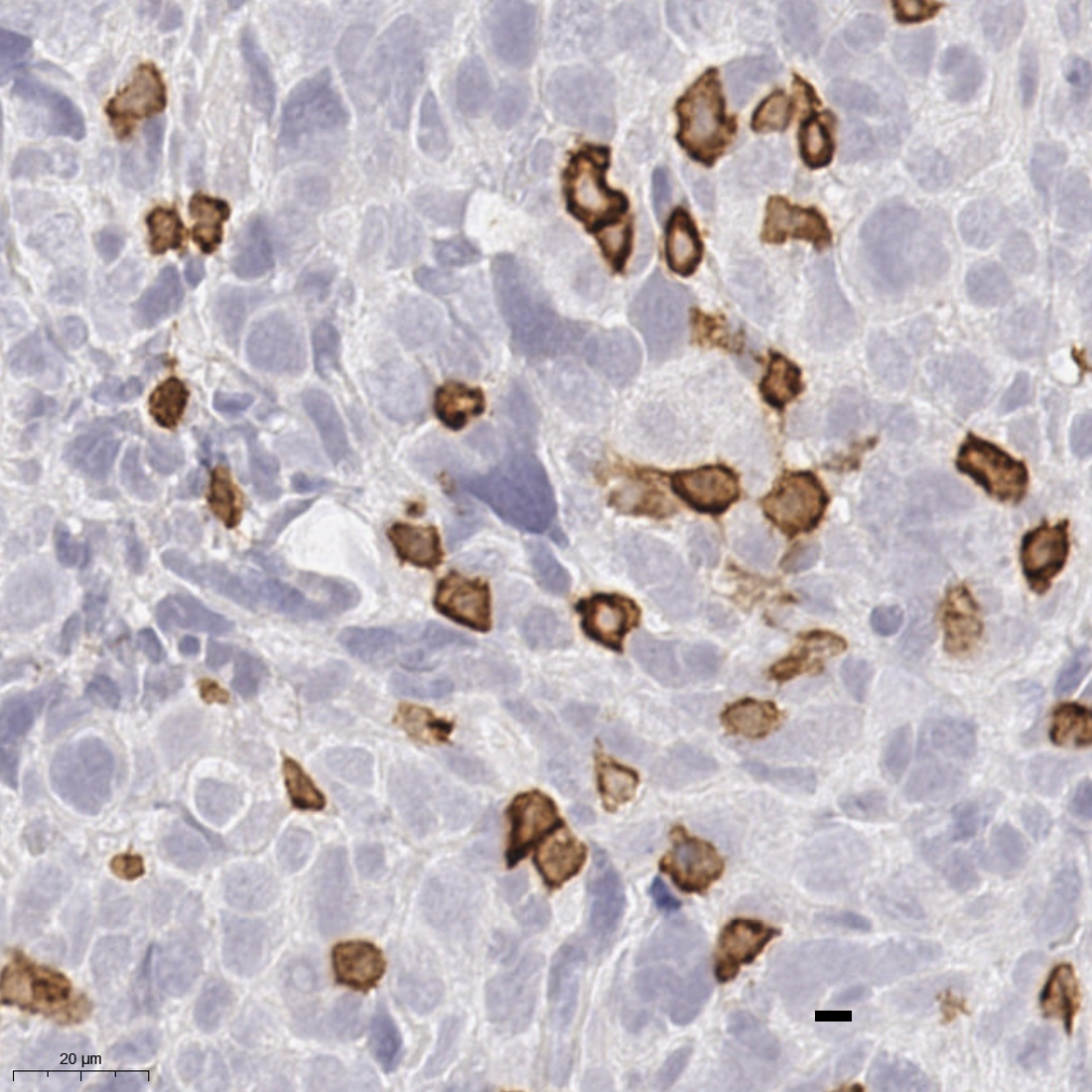

Supplement: Supplementary file 8 — Source data Fig. 6 [file 44321_2024_94_MOESM8_ESM.zip › Figure 6/6A Image data micr. image/Brca1def Flt1i + Tal.tif]

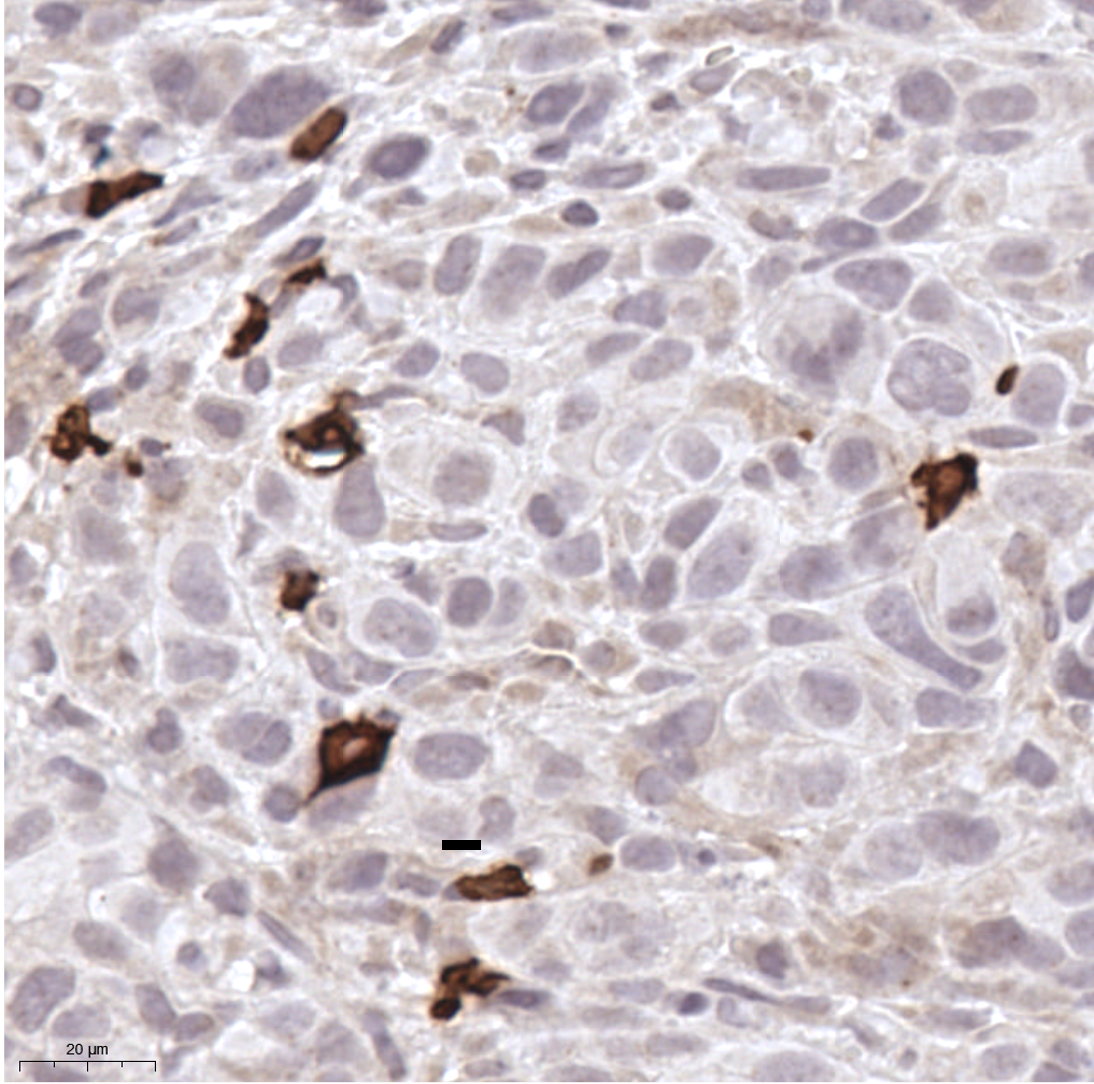

Supplement: Supplementary file 8 — Source data Fig. 6 [file 44321_2024_94_MOESM8_ESM.zip › Figure 6/6A Image data micr. image/Bard1def Flt1i + Tal.tif]

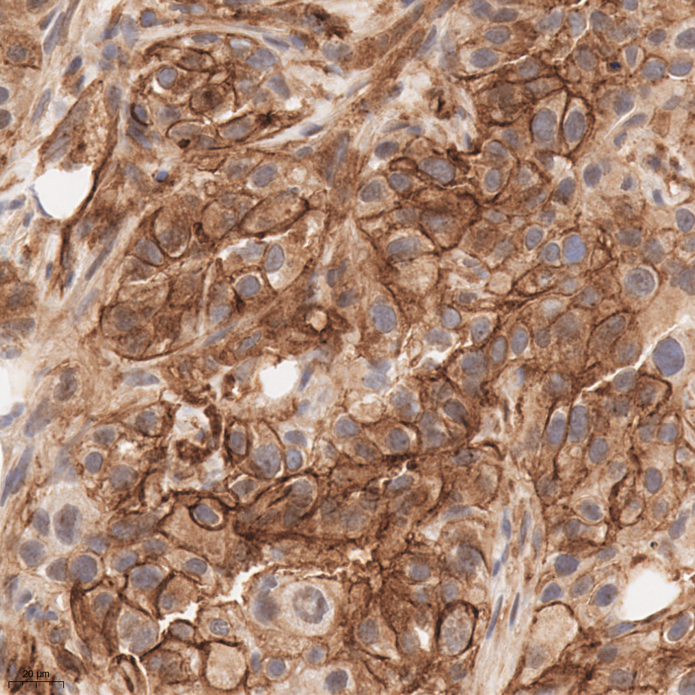

Supplement: Supplementary file 9 — Source data Fig. 7 [file 44321_2024_94_MOESM9_ESM.zip › Figure 7/7B Image data micr. image/pFLT1 High.tif]

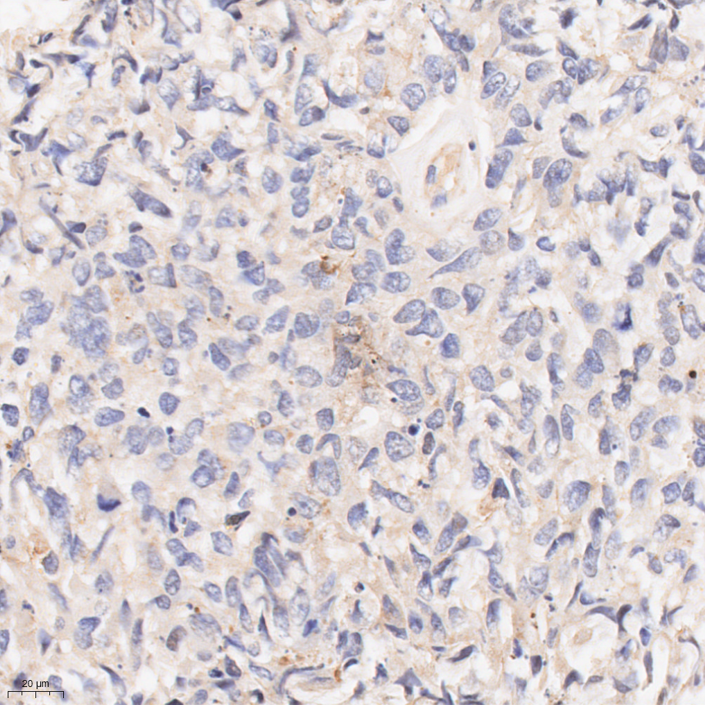

Supplement: Supplementary file 9 — Source data Fig. 7 [file 44321_2024_94_MOESM9_ESM.zip › Figure 7/7B Image data micr. image/pFLT1 Low.tif]

Pre Samples : pVEGFR1

p.val = 0.012 & Chisq = 6.325

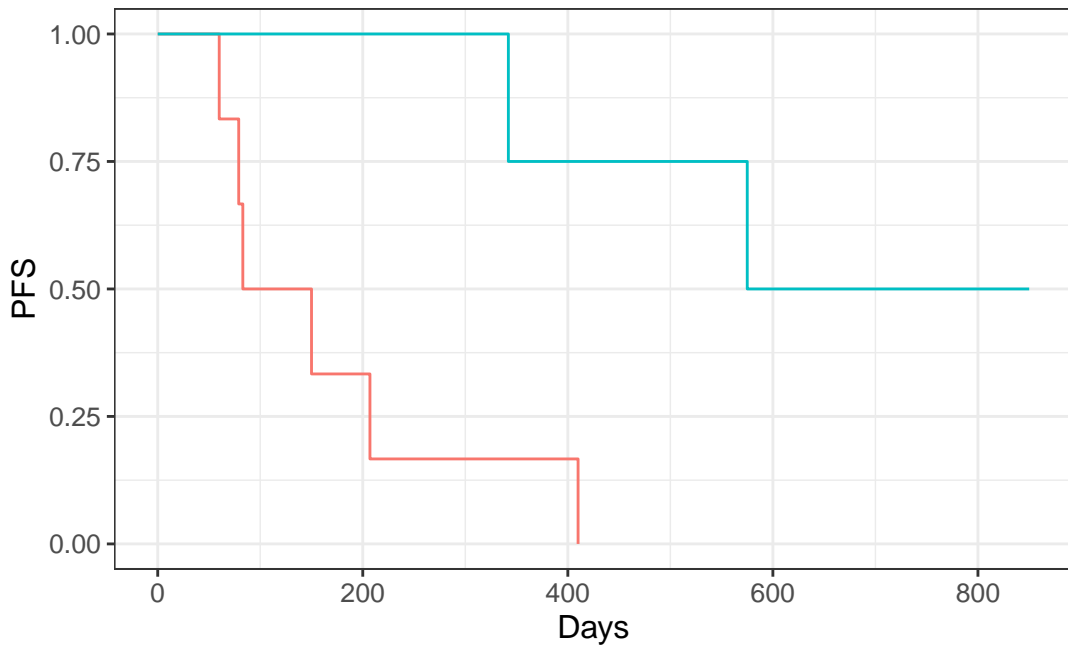

— high — low

high

|         |   |   |   |   |   |
|---------|---|---|---|---|---|
| At Risk | 6 | 2 | 1 | 0 | 0 |
| Events  | 0 | 4 | 5 | 6 | 6 |

low

|         |   |   |   |   |   |
|---------|---|---|---|---|---|
| At Risk | 4 | 4 | 3 | 2 | 2 |
| Events  | 0 | 0 | 1 | 2 | 2 |

Supplement: Supplementary file 9 — Source data Fig. 7 [file 44321_2024_94_MOESM9_ESM.zip › Figure 7/7C Numerical data (survival curve)/Survival curve (provided by our statician collaborator).pdf]
