# Supplementary material for: FLT1 activation in cancer cells promotes PARP-inhibitor resistance in breast cancer
Source: EMBO Mol Med. 2024 Jul 2;16(8):8. doi: 10.1038/s44321-024-00094-2 (PMC11319505; doi:10.1038/s44321-024-00094-2)
Supplement: Supplementary file 10 — Expanded View Figures [file 44321_2024_94_MOESM10_ESM.pdf]

Expanded View Figures

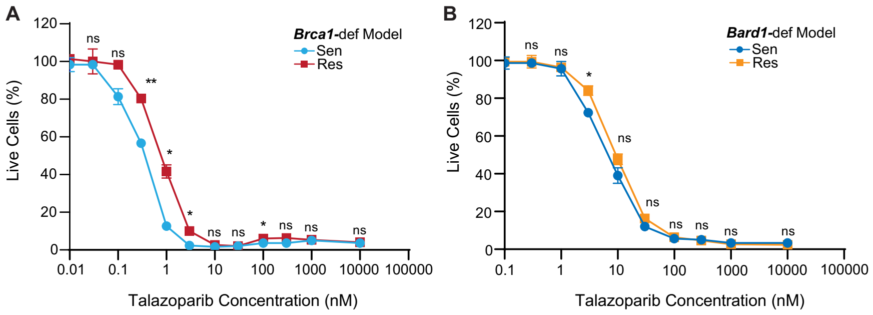

Figure EV1. Modest differences in talazoparib sensitivity observed in vitro compared to in-vivo settings (Fig. 1) in *Brca1*-def and *Bard1*-def breast tumor cells.

(**A**, **B**) In vitro cell viability assay comparing the talazoparib sensitivity between talazoparib-sensitive ("Sen") *Brca1*-def and *Bard1*-def breast tumor cells and their talazoparib-resistant ("Res") derivative lines. Cell viability values are normalized to the DMSO-treated control (considered as 100% live cells) and presented as mean values ± SEM. *P* values were determined by a two-tailed, unpaired, Welch's test. For the *Brca1*-def model, $n = 3$ for each concentration tested, ** at 0.3 nM indicates $P = 0.0028$, * at 1 nM indicates $P = 0.0104$, * at 3 nM indicates $P = 0.0104$, and * at 100 nM indicates $P = 0.0357$. For the *Bard1*-def model, $n = 3$ for each tested concentration, and * at 3.0 nM indicates $P = 0.0256$. ns; not significant.

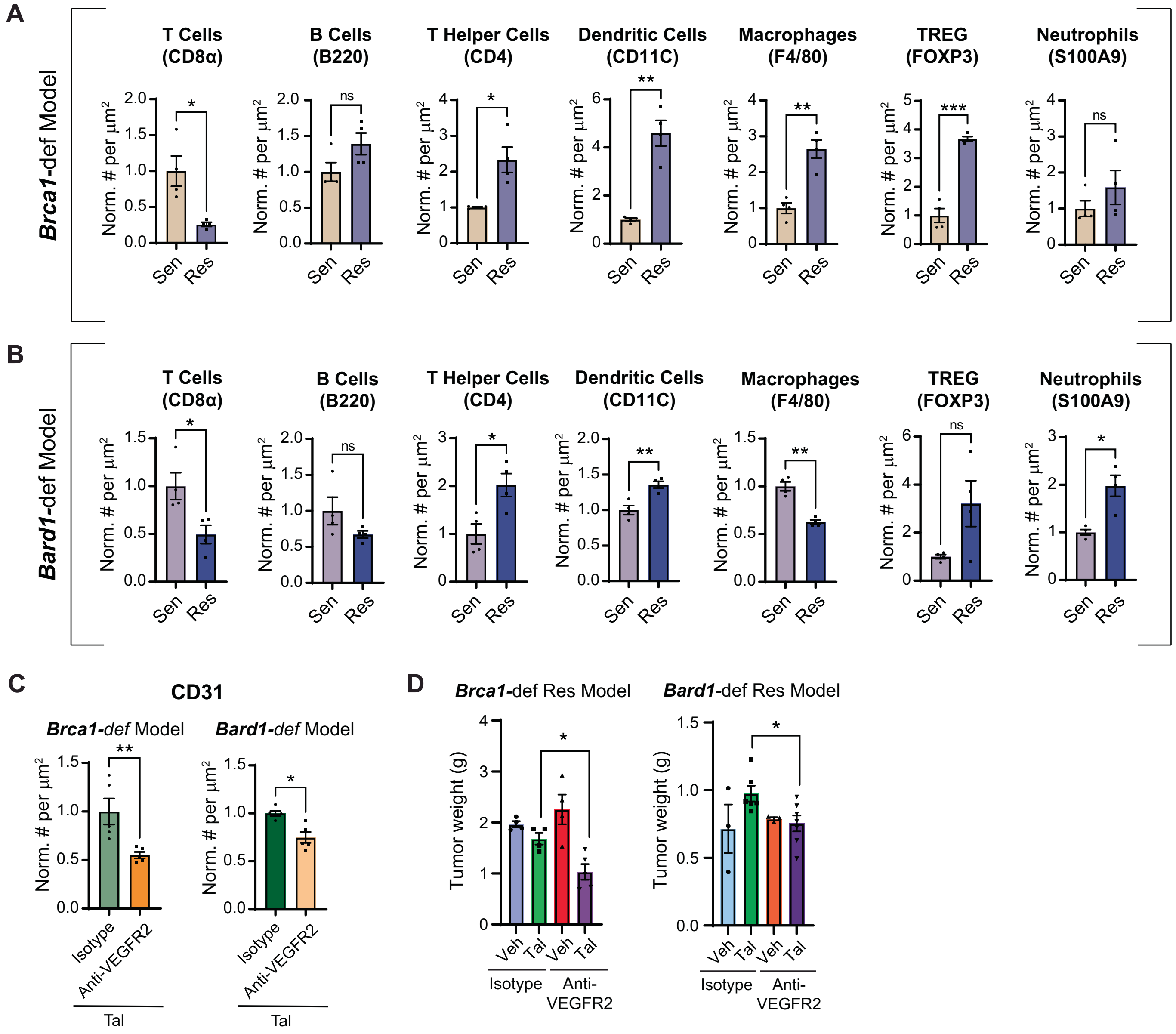

Figure EV2. Quantification of angiogenesis and immune-cell composition in *Brca1*-def and *Bard1*-def mutant breast tumors by immunostaining analysis.

(**A**, **B**) Immunostained tumor sections from Fig. 1 were quantified using automated QuPath software to identify positively stained cells. Data were presented as mean values ± SEM. *P* values were determined by a two-tailed, unpaired, Welch's test. $n = 4$ mice for talazoparib-sensitive ('Sen") and -resistant ("Res") groups from both models. *P* values are listed in parentheses following the protein analyzed. For the *Brca1*-def model in (**A**): CD8α (0.0375), CD4 (0.0329), CD11C (0.0061), F4/80 (0.0027), and FOXP3 (0.0007). For the *Bard1*-def model in (**B**), CD8α (0.0293), CD4 (0.0186), CD11C (0.0048), F4/80 (0.0016), FOXP3 (ns), and S100A9 (0.0182). ns; not significant. (**C**) Quantitation of immunostained Res tumor sections using antibodies against CD31 (see treatments in Fig. 2I–K) using automated QuPath software to identify positively stained cells. $n = 5$ mice from both groups from both models. Data were presented as mean values ± SEM. *P* values were determined by a two-tailed, unpaired, Mann–Whitney test. ** indicates $P = 0.0079$ for the *Brca1*-def model, and * indicates 0.0159 for the *Bard1*-def model. (**D**) Tumor weights from Fig. 2J were plotted after collection at endpoint. For the *Brca1*-def model, $n = 4$ tumors for the Isotype + Veh group, $n = 4$ tumors for the Isotype + Tal group, $n = 4$ tumors for the Anti-VEGFR2 + Veh group, and $n = 5$ tumors for the anti-VEGFR2 + Tal group. For the *Bard1*-def model, $n = 3$ tumors for Isotype + Veh group, $n = 6$ tumors for Isotype + Tal group, $n = 3$ tumors for anti-VEGFR2 + Veh group, and $n = 7$ tumors for anti-VEGFR2 + Tal group. Data were presented as mean values ± SEM. *P* values were determined by a two-tailed, unpaired, Student's *t*-test, comparing endpoint tumor weights between the Isotype + Tal and anti-VEGFR2 + Tal groups. * indicates $P = 0.0141$ for the *Brca1*-def model, and * indicates $P = 0.0222$ for the *Bard1*-def model.

 © The Author(s)

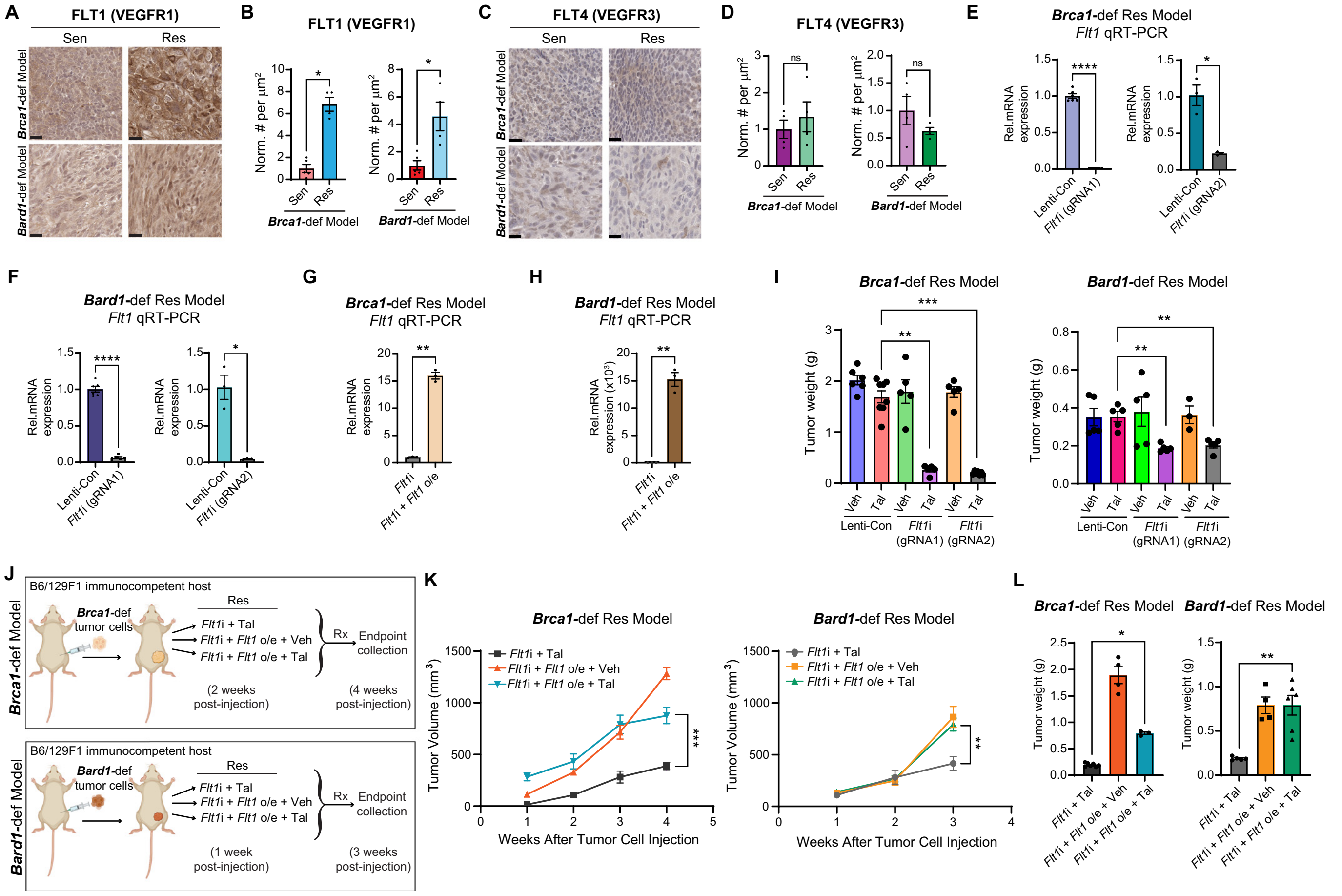

A
FLT1 (VEGFR1)
Sen
Res
Brca1-def Model
Bard1-def Model
B
FLT1 (VEGFR1)
Norm. # per µm²
Sen
Res
Brca1-def Model
Bard1-def Model
C
FLT4 (VEGFR3)
D
FLT4 (VEGFR3)
ns
E
Brca1-def Res Model
Flt1 qRT-PCR
Rel.mRNA expression
Lenti-Con
Flt1i (gRNA1)
Flt1i (gRNA2)
F
Bard1-def Res Model
Flt1 qRT-PCR
G
Brca1-def Res Model
Flt1 qRT-PCR
Flt1i
Flt1i + Flt1 o/e
H
Bard1-def Res Model
Flt1 qRT-PCR
Rel.mRNA expression (x10³)
I
Brca1-def Res Model
Bard1-def Res Model
Tumor weight (g)
Veh
Tal
Lenti-Con
Flt1i (gRNA1)
Flt1i (gRNA2)
J
B6/129F1 immunocompetent host
Brca1-def tumor cells
Res
Flt1i + Tal
Flt1i + Flt1 o/e + Veh
Flt1i + Flt1 o/e + Tal
Rx
Endpoint collection
(2 weeks post-injection)
(4 weeks post-injection)
Brca1-def Model
B6/129F1 immunocompetent host
Bard1-def tumor cells
(1 week post-injection)
(3 weeks post-injection)
Bard1-def Model
K
Brca1-def Res Model
Bard1-def Res Model
Tumor Volume (mm³)
Weeks After Tumor Cell Injection
L
Brca1-def Res Model
Bard1-def Res Model
Tumor weight (g)
Flt1i + Tal
Flt1i + Flt1 o/e + Veh
Flt1i + Flt1 o/e + Tal

Figure EV3. FLT1 promotes PARPi-resistance in the *Brca1*-def and *Bard1*-def breast cancer models.

(**A**) Representative images of IHC for total FLT1 expression in tumor sections from the mice described in Fig. 1. Scale bars, 20 μm. (**B**) Immunostained sections from (**A**) were quantified using automated QuPath software to identify positively stained cells. *n* = 5 talazoparib-sensitive ("Sen") tumors, and *n* = 4 talazoparib-resistant ("Res") tumors from both *Brca1*-def and *Bard1*-def models. Data were presented as mean values ± SEM. *P* values were determined by a two-tailed, unpaired, Mann–Whitney test. * indicates *P* = 0.0159 for both models. (**C**), Representative images of IHC for FLT4 in tumor sections from Fig. 1. Scale bars, 20 μm. (**D**) Immunostained sections from (**C**) were quantified using automated QuPath software to identify positively stained cells. *n* = 4 Sen and Res tumors for both *Brca1*-def and *Bard1*-def models. Data were presented as mean values ± SEM. *P* values were determined by a two-tailed, unpaired, Welch's test. ns; not significant. (**E**) qRT-PCR results of *Flt1* repression of the indicated groups in the *Brca1*-def model for both gRNAs. *n* = 6 (consisting of two independent experiments for each triplicate testing) for both Lenti-Con and *Flt1*i for gRNA1 and *n* = 3 (one triplicate testing) for both Lenti-Con and *Flt1*i for gRNA2. Data were presented as mean values ± SEM. *P* values were determined by a two-tailed, unpaired, Welch's test: **** indicates *P* < 0.0001 for gRNA1 and * indicates 0.0285 for gRNA2. (**F**) qRT-PCR results of *Flt1* repression of the indicated groups in the *Bard1*-def model. *n* = 6 (two independent triplicate testing) for both Lenti-Con and *Flt1*i for gRNA1 and *n* = 3 (one triplicate testing) for both Lenti-Con and *Flt1*i for gRNA2. Data were presented as mean values ± SEM. *P* values were determined by a two-tailed, unpaired, Welch's test: **** indicates *P* < 0.0001 for gRNA1 and * indicates *P* = 0.0273 for gRNA2. (**G**) qRT-PCR results of *Flt1* expression of the indicated groups in the *Brca1*-def model. *n* = 3 (one triplicate testing) for *Flt1*i and *Flt1*i + *Flt1* overexpression ("o/e") groups. Data were presented as mean values ± SEM. *P* values were determined by a two-tailed, unpaired, Welch's test: ** indicates *P* = 0.0016. (**H**) qRT-PCR results of *Flt1* expression of the indicated groups in the *Bard1*-def model. *n* = 3 (one triplicate testing) for *Flt1*i and *Flt1*i + *Flt1* o/e groups. Data were presented as mean values ± SEM. *P* values were determined by a two-tailed, unpaired, Welch's test: ** indicates *P* = 0.0067. (**I**) Tumor weights from Fig. 3D were plotted at endpoint. For the *Brca1*-def model, *n* = 6 tumors for Lenti-Con + Veh, *n* = 8 tumors for Lenti-Con + Tal, *n* = 5 tumors for *Flt1*i (gRNA1) + Veh or Tal and *Flt1*i (gRNA2) + Veh, and *n* = 7 tumors for *Flt1*i (gRNA2) + Tal treatment groups. For the *Bard1*-def model, *n* = 5 tumors for Lenti-Con + Veh or Tal, *Flt1*i (gRNA1) + Veh or Tal, and *Flt1*i (gRNA2) + Tal, and *n* = 3 tumors for *Flt1*i (gRNA2) + Veh. Data were presented as mean values ± SEM. *P* values were determined by a two-tailed, unpaired, Mann–Whitney test, comparing endpoint tumor weights between Lenti-Con + Tal and *Flt1*i (gRNA1 or gRNA2) + Tal groups. For the *Brca1*-def model, ** indicates *P* = 0.0016 between Lenti-Con + Tal and *Flt1*i (gRNA1) + Tal and *** indicates *P* = 0.0003 between Lenti-Con + Tal and *Flt1*i (gRNA2) + Tal. For the *Bard1*-def model, ** indicates *P* = 0.0079 between Lenti-Con + Tal and *Flt1*i (gRNA1 or gRNA2) + Tal. (**J**) Schematic representation of the experiment designed to test whether *Flt1* re-expression rescues talazoparib-resistance in *Brca1*-def and *Bard1*-def mammary tumors with *Flt1* repression. The generation of *Brca1*- and *Bard1*-def cancer cells were described in Fig. 3. To stably re-express *Flt1*, we transduced *Flt1*-repressed cells with lentiviral particles carrying *Flt1* cDNA. For the *Brca1*-def model, randomized mice received either vehicle ("Veh") or talazoparib ("Tal") treatment starting at 2 weeks after tumor-cell injection and were euthanized at 4 weeks following injection. For the *Bard1*-def model, randomized mice received treatment at 1 week after tumor-cell injection and were euthanized at 3 weeks following injection. (**K**) Tumor growth curves comparing *Flt1*i + Tal and *Flt1*i-*Flt1* o/e + Veh or Tal. For the *Brca1*-def model, *n* = 7 mice for *Flt1*i + Tal, *n* = 4 mice for *Flt1*i-*Flt1* + o/e + Veh, and *n* = 3 mice for *Flt1*i-*Flt1* + o/e + Tal. For the *Bard1*-def model, *n* = 5 mice for *Flt1*i + Tal, *n* = 4 mice for *Flt1*i-*Flt1* + o/e + Veh, and *n* = 6 mice for *Flt1*i-*Flt1* + o/e + Tal. Data were presented as mean values ± SEM. *P* values were determined with a one-way ANOVA test, comparing endpoint tumor volumes between the *Flt1*i + Tal and *Flt1*i-*Flt1* + o/e + Tal groups. For the *Brca1*-def model, *** at 4 weeks indicates *P* = 0.0001 and for the *Bard1*-def model, ** at 3 weeks indicates *P* = 0.0074. (**L**) Tumor weights from K were plotted at endpoint. For the *Brca1*-def model, *n* = 7 tumors for *Flt1*i + Tal, *n* = 4 tumors for *Flt1*i-*Flt1* + o/e + Veh, and *n* = 3 tumors for *Flt1*i-*Flt1* + o/e + Tal. For the *Bard1*-def model, *n* = 5 tumors for *Flt1*i + Tal, *n* = 4 tumors for *Flt1*i-*Flt1* + o/e + Veh, and *n* = 6 tumors for *Flt1*i-*Flt1* + o/e + Tal. Data were presented as mean values ± SEM. *P* values were determined by a two-tailed, unpaired, Mann–Whitney test, comparing endpoint tumor weights between the *Flt1*i + Tal and *Flt1*i-*Flt1* + o/e + Tal groups. For the *Brca1*-def model, * indicates *P* = 0.0167 and for the *Bard1*-def model, ** indicates *P* = 0.0043.

 © The Author(s)

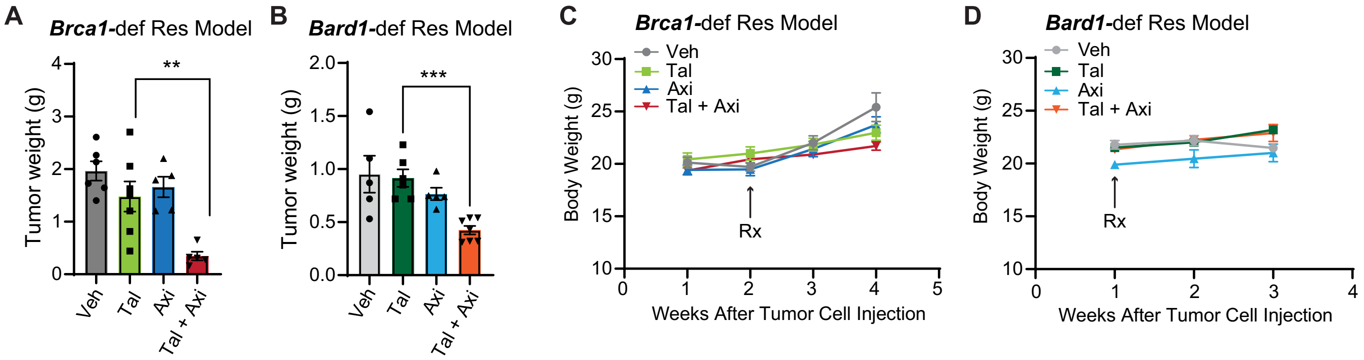

Figure EV4. Tumor and body weight analysis of mice treated with vehicle ("Veh"), talazoparib ("Tal"), and/or axitinib ("Axi").

(**A**) Tumor weights from Fig. 4B were plotted at endpoint. $n = 6$ Veh-treated tumors, $n = 7$ Tal-treated tumors, $n = 5$ Axi-treated tumors, and $n = 5$ tumors treated with Tal + Axi. Data were presented as mean values ± SEM. *P* values were determined by a two-tailed, unpaired, Mann–Whitney test, comparing endpoint tumor weights between the Tal and Tal + Axi groups. ** indicates $P = 0.0051$. (**B**) Tumor weights from Fig. 4E were plotted after collection at endpoint. $n = 5$ Veh-treated tumors, $n = 6$ Tal-treated tumors, $n = 5$ Axi-treated tumors, and $n = 7$ tumors treated with Tal + Axi. Data were presented as mean values ± SEM. *P* values were determined by a two-tailed, unpaired Mann–Whitney test comparing endpoint tumor weights between the Tal and Tal + Axi groups. *** indicates $P = 0.0006$. (**C**, **D**) Body weight from treatment initiation until endpoint from Fig. 4A, D for each experiment. For the *Brca1*-def model, $n = 6$ Veh-treated tumors, $n = 7$ Tal-treated tumors, $n = 5$ Axi-treated tumors, and $n = 5$ tumors treated with Tal + Axi. For the *Bard1*-def model, $n = 5$ Veh-treated tumors, $n = 6$ Tal-treated tumors, $n = 5$ Axi-treated tumors, and $n = 7$ tumors treated with Tal + Axi.

© The Author(s)

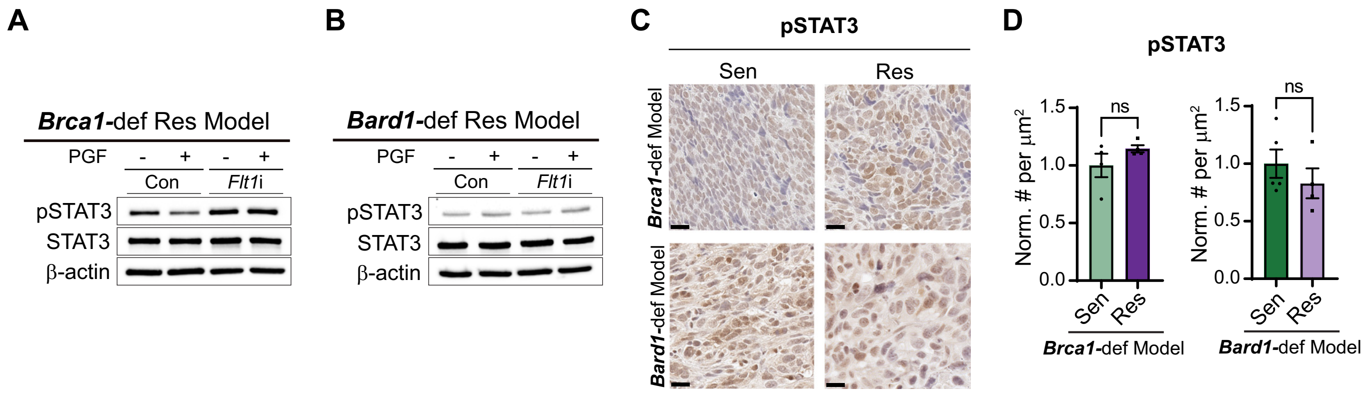

Figure EV5. Quantitation of phosphorylated STAT3 (pSTAT3) levels in *Brca1*-def and *Bard1*-def breast tumor cells and tumor tissue sections.

(**A**, **B**) Immunoblot analysis was performed using antibodies against pSTAT3, STAT3 and β-actin (loading control) using lysates from *Flt1*-expressing (Con) and -deficient (*Flt1*i), talazoparib-resistant ("Res") *Brca1* and *Bard1*-def tumor cells, treated with 50 ng/mL of mouse PGF protein that were used in Fig. 5A, B. (**C**) Representative images of IHC for pSTAT3 staining in tumor sections from the mice described in Fig. 1 comparing talazoparib-sensitive ("Sen") tumors to talazoparib-resistant ("Res") tumors. Scale bars, 20 μm. (**D**) Immunostained sections from (**C**) were quantified using automated QuPath software to identify positively stained cells. For the *Brca1*-def model, $n = 4$ tumors for both Sen and Res. For the *Bard1*-def model, $n = 5$ Sen tumors, and $n = 4$ Res tumors. Data were presented as mean values ± SEM. *P* values were determined by a two-tailed, unpaired, Mann–Whitney test. ns; not significant.

 © The Author(s)
